# Curcumin regulates autophagy through SIRT3-SOD2-ROS signaling pathway to improve quadriceps femoris muscle atrophy in KOA rat model

**DOI:** 10.1038/s41598-024-58375-2

**Published:** 2024-04-08

**Authors:** Hua Ye, Yi Long, Jia-Ming Yang, Yan-Lin Wu, Ling-Yan Dong, Yan-Biao Zhong, Yun Luo, Mao-Yuan Wang

**Affiliations:** 1 Department of Rehabilitation Medicine, First Affiliated Hospital of Gannan Medical University, 128 Jinling Road, Zhanggong District, Ganzhou, 341000 Jiangxi China; 2 Ganzhou Intelligent Rehabilitation Technology Innovation Center, Ganzhou, Jiangxi China; 3 Ganzhou Key Laboratory of Rehabilitation Medicine, Ganzhou, Jiangxi China

**Keywords:** Knee osteoarthritis, Quadriceps femoris muscle, Curcumin, Autophagy, Experimental models of disease, Inflammation

## Abstract

Knee osteoarthritis (KOA) usually leads to quadriceps femoris atrophy, which in turn can further aggravate the progression of KOA. Curcumin (CUR) has anti-inflammatory and antioxidant effects and has been shown to be a protective agent for skeletal muscle. CUR has been shown to have a protective effect on skeletal muscle. However, there are no studies related to whether CUR improves KOA-induced quadriceps femoris muscle atrophy. We established a model of KOA in rats. Rats in the experimental group were fed CUR for 5 weeks. Changes in autophagy levels, reactive oxygen species (ROS) levels, and changes in the expression of the Sirutin3 (SIRT3)-superoxide dismutase 2 (SOD2) pathway were detected in the quadriceps femoris muscle of rats. KOA led to quadriceps femoris muscle atrophy, in which autophagy was induced and ROS levels were increased. CUR increased SIRT3 expression, decreased SOD2 acetylation and ROS levels, inhibited the over-activation of autophagy, thereby alleviating quadriceps femoris muscle atrophy and improving KOA. CUR has a protective effect against quadriceps femoris muscle atrophy, and KOA is alleviated after improvement of quadriceps femoris muscle atrophy, with the possible mechanism being the reduction of ROS-induced autophagy via the SIRT3-SOD2 pathway.

## Introduction

Knee osteoarthritis (KOA) is a very common chronic musculoskeletal disease that widely affects many people around the world^1^. The fundamental hallmark of KOA is the degradation of intra-articular cartilage^2^, but it is also characterized by changes in the muscles around the joint, such as weakness and atrophy of the quadriceps femoris muscle being the most common^3–5^. The quadriceps femoris muscle is not only an active muscle that produces knee extension movements but also provides knee stability. Quadriceps femoris muscle weakness and atrophy can further worsen the development of KOA^6^. Inflammation of the KOA leads to presynaptic reflex inhibition and decreases alpha motor neuron activity, resulting in quadriceps femoris muscle atrophy and weakness^7,8^. Joint pain and reduced mobility in patients with KOA can lead to the development of disuse atrophy in the quadriceps femoris muscle^9^. Atrophy and weakness of the quadriceps femoris muscle lead to biomechanical alterations and overloading of the knee joint, accelerating the destruction of cartilage^10^. Therefore, a vicious cycle of knee degeneration and skeletal muscle atrophy is formed. Improving the atrophy of the quadriceps femoris muscle can alleviate the progression of KOA and thus break this vicious cycle. Exercise training to strengthen the quadriceps femoris muscle has been shown to improve KOA^11^. Most of the current studies on the muscles of KOA patients are on muscle function, and there are fewer studies on the structural and cellular molecular changes in the muscles. More studies are needed to explore the molecular mechanisms of quadriceps atrophy, which can help to promote quadriceps regeneration in KOA patients and also help to further optimize KOA treatment protocols.

Muscle atrophy is caused by an imbalance in protein synthesis and degradation. Two major proteolytic systems, the ubiquitin–proteasome system and the autophagy lysosome system, are activated during skeletal muscle atrophy^12^. Autophagy is required in muscle to maintain muscular mass, either inhibition or excessive activation of autophagy can lead to atrophy in skeletal muscle^13–15^. However, how KOA-induced quadriceps atrophy autophagy changes is unknown. Excessive accumulation of reactive oxygen species (ROS) can lead to atrophy of skeletal muscle^16–18^. ROS can induce autophagy^18^. Sirutin3 (SIRT3) is a deacetylase located primarily in mitochondria. It regulates a variety of mitochondrial functions, including energy metabolism and anti-oxidative stress^19^. SIRT3 can bind directly to superoxide dismutase 2 (SOD2) and then deacetylate it, thereby increasing the activity of SOD2 and significantly affecting ROS homeostasis and autophagic flux^20,21^.

Curcumin (CUR) is a natural polyphenolic compound extracted from turmeric root. It mainly has anti-inflammatory and antioxidant properties^22,23^. It has been shown that CUR can ameliorate exercise-induced muscle damage, which is primarily achieved by decreasing the inflammatory response and increasing antioxidant capacity^24–27^. CUR also can reduce skeletal muscle mitochondrial damage and improve muscle atrophy in chronic obstructive pulmonary disease rats by activating SIRT3^28^. CUR can effectively inhibit palmitate-induced inflammation in skeletal muscle cells as well as scavenge intracellular ROS^29^. In addition, CUR protects heat-induced skeletal muscle damage in a dose-dependent manner by preventing mitochondrial dysfunction, ROS increase, and apoptosis^30^. CUR can effectively improve muscle disuse muscular atrophy by activating the deacetylase sirtuin-1^31,32^. There were also numerous studies confirming that CUR treats sarcopenia by exerting anti-inflammatory and antioxidant effects^33–35^. CUR not only has a protective effect on skeletal muscle, it also has a therapeutic effect on KOA^36–38^. It has been shown that CUR can attenuate cartilage damage in KOA rats by inhibiting the inflammatory response through acting on the NF-κB signaling pathway^39,40^. CUR may also prevent chondrocyte damage by inhibiting oxidative stress^41^. It has also been shown that CUR can act on mitochondrial autophagy to exert chondroprotective effects in KOA rats^42^. There are no relevant studies on the role of CUR in KOA-induced quadriceps femoris muscle atrophy.

In this study, we established KOA model rats to explore the mechanism of CUR in KOA-induced quadriceps femoris muscle atrophy. Based on the results of previous studies, we hypothesized that CUR could regulate autophagy to ameliorate quadriceps femoris muscle atrophy by activating SIRT3, deacetylating SOD2, and increasing the activity of SOD2 to scavenge ROS. As the quadriceps femoris muscle atrophy improved, the knee joint returned to normal biomechanics and weight bearing. As well as the therapeutic effect of CUR on KOA. As a result, KOA can be better treated.

## Materials and methods

### Experimental animals

Thirty-two 5-week-old healthy male Sprague Dawley rats (purchased from Hunan Silaike Jingda Experimental Animal Co., Ltd, China)^43^. After 1 week of acclimatization feeding, the rats were randomly divided into four groups: Control group (Control), Sham group (Sham), KOA group (KOA), and KOA + CUR group (KOA + CUR), with eight rats assigned to each group. The experiments were conducted in accordance with relevant guidelines and regulations. The study complied with ARRIVE guidelines.

### Modeling and medication

**Figure 1:**
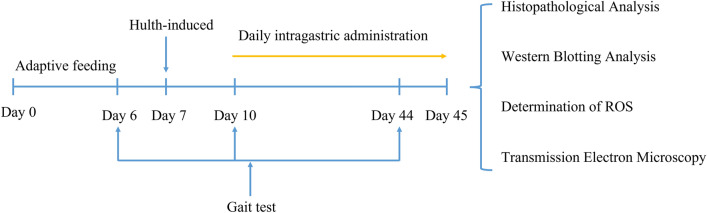
The schematic diagram of the experimental procedures. Gait analysis was performed the day before the Hulth procedure, 3 days after the procedure, and 35 days after the intervention. CUR treatment was started on the third day after the Hulth procedure, once a day for 35 days. Knee joints and quadriceps femoris muscle tissues were collected at the end of instillation and used for subsequent experiments.

The Hulth method was used to establish a model of KOA^44^. After 12 h of fasting, an intraperitoneal injection of 18% urane (94,300, Sigma) at a dose of 0.45 ml/100 g was administered, followed by immobilization in the supine position. The right knee was incised to expose the joint cavity and subsequently the medial collateral ligament, anterior and posterior cruciate ligaments were severed and the medial meniscus was outlined without any damage to the articular cartilage surface. This was confirmed by the drawer test^44^. Rats in the KOA group and rats in the KOA + CUR group underwent Hulth surgery. Rats in the control group did not undergo any intervention. Sham group rats were sutured after opening the joint cavity without damaging the ligaments or medial meniscus. Rats in the KOA group were fed with a 0.5% solution of sodium carboxymethyl cellulose (2 ml/day, 419273, Sigma) for 5 weeks. Rats in the KOA + CUR group were fed with CUR (150 mg/kg/day, SR3294, HARVEYBIO, China), which was dissolved in 2 ml 0.5% sodium carboxymethyl cellulose. The experimental procedure was shown in Fig. 1.

### Sacrifice and sample collection

After 5 weeks, the rats in each group were anesthetized by intraperitoneal injection of 18% urane (0.45 ml/100 g) and subsequently euthanized by cervical dislocation. The quadriceps femoris muscle and knee joints of the right hind leg of each group of rats were obtained, rapidly frozen in liquid nitrogen, and subsequently stored at – 80 ℃. In addition, the quadriceps femoris muscle and knee joints for histological analysis were fixed in a 4% paraformaldehyde solution (BL539A, Biosharp Bio Inc, China).

### Gait test

We used the CatWalk XT automated gait analysis system to analyze the gait of each group of rats (Noldus Information Technology, Netherlands, https://www.noldus.com/catwalk-xt)^45,46^. The software name is CatWalk™ XT and version is 10.6. It consists of a 1.3-m-long horizontal glass panel covered with a movable tunnel that produces a dim light on the walkway. Gait analysis was performed before the establishment of the KOA model, 3 days after surgery, and 5 weeks after intervention. The rats were placed at the beginning of the walkway and ran through the glass panels to the end. The run criteria was set to a maximum speed variation of 60% and a minimum duration of 0.5 s^47^. The gait analysis results of rats were sent to the computer and automatically analyzed by the CatWalk^TM^ XT software. The rats must perform at least three uninterrupted runs that meet the requirements of the CatWalk^TM^ XT analysis. A manual review of the automated tracking identifications was conducted to improve the quality of the data. We used CatWalk^TM^ XT software to analyze the maximum contact area, standing length, swing length, and mean pressure of the right hind foot of 32 rats.

### Histopathological analysis

After 48 h of fixation in paraformaldehyde, the knee joints were placed in Ethylenediaminetetraacetic acid decalcification solution (G1105, Servicebio, China) in a thermostatic shaker (ZHPW-250, BLabotery) for thermostatic shaker decalcification (30 ℃), and the decalcification solution was changed once a week, and the procedure was continued for 3 weeks. During this procedure, the bone is tested with a needle to see if it can be punctured. Decalcification was successful when the bones became soft and flexible. Afterwards, the knee is cut in the sagittal plane, dehydrated in gradient alcohol, paraffin-embedded, and sectioned (5 μm). The sections were then stained for hematoxylin–eosin (H&E), (G1003, Servicebio, China) and Safranin-O/Fast Green staining (G1053, Servicebio, China)^48,49^. The Osteoarthritis Research Society International (OARSI) histopathological grading system assesses changes in the structural cartilage of the knee joint^50,51^. Injury grades ranging from 0 to 6 represent the depth of progression of cartilage damage. Injury stages from 0 to 4 represent the degree of cartilage involvement. The final score combined grade and stage (score range 0–24). As in previous studies, two independent evaluators scored the knee to avoid evaluator bias^50,52^.

The quadriceps femoris muscle was removed from the fixative and gradient dehydrated, paraffin embedded, sectioned (5 μm), and then subjected to conventional H&E staining. Muscle fiber cross-sectional area (CSA) of rat muscle fibers were calculated using ImageJ software (National Institutes of Health, Stapleton, NY, USA)^53^.

### Western blotting analysis

Quadriceps femoris muscle was collected from each group of rats for Western blot analysis. Protein was extracted from 1 g of quadriceps femoris muscle tissue using a 1.5 ml RIPA reaction solution supplemented with protease inhibitors. The samples were left at 100℃ for 10 min and then stored in a − 20 °C freezer. Each sample was separated by sodium dodecyl sulfate–polyacrylamide gel electrophoresis (SDS-PAGE) and 30 μg transferred to the polyvinylidene difluoride (PVDF) membrane. We cut the PVDF membrane while retaining sufficient width and length to allow different positions of the PVDF membrane to bond with different primary antibodies. Then blotted with primary antibodies for LC3 (1:1000, ab192890, Abcam)^54^, P62 (1:1000, ab109012, Abcam)^55^, SIRT3 (1:500, #5490, CST)^56^, SOD2 (1:1000, #13141, CST)^57^, acetylated SOD2 (1:1000, ab137037, Abcam)^58^ and GAPDH (1:2000, 60004-1-Ig, Proteintech)^59^ overnight at 4 °C. Then, incubate with the secondary antibody for 1.5 h at room temperature. Protein bands were displayed using an ECL Western blotting substrate (PE0010-2, Solarbio). Finally, protein bands were quantified using ImageJ software^60^.

### Determination of ROS and SOD2 activity assay

Make a homogenate of 50 mg from fresh quadriceps femoris muscle with 1 mL of buffer, use the homogenate and centrifuge 4 °C at 1000×*g* for 10 min. The supernatant was collected and incubated with an ROS probe (BB470515, BestBio, China) for 30 min at 37 °C in the dark^61,62^. ROS levels were quantified under fluorescent zymography (Bio-Tek Instruments, USA) with an excitation wavelength of 480 nm and an emission wavelength of 615 nm.

SOD2 activity (U, in mg of protein) in rat quadriceps femoris muscle was determined using a superoxide dismutase (SOD) typing assay kit (A001-2-2, Jiancheng Biotech, Nanjing, China)^63^.

### Transmission electron microscopy

The quadriceps femoris muscle was stripped from the rat, cut into 1 mm^3^ size and quickly fixed in 2.5% glutaraldehyde at 4 ℃ for 48 h. The tissue was then removed and rinsed with 0.1 M phosphate buffer (pH 7.4). Dehydration was performed at room temperature, followed by resin penetration and embedding at 37 °C. Sections (60 nm) were performed after polymerization using an ultrathin microtome (Leica UC7, Leica). Staining was then performed as follows: Soak in a 2% uranyl acetate saturated alcohol solution for 8 min to avoid light staining, then rinse 3 times with 70% ethanol and 3 times with ultrapure water. 2.6% lead citrate avoided CO_2_ staining for 8 min, then rinsed 3 times with ultrapure water. After drying on filter paper, the copper-beryllium grid was placed on a grid plate and dried overnight at room temperature. Finally, the sections were observed under a transmission electron microscope (HT7800/HT7700, HITACHI) and images were captured^64^.

### Statistical analysis

All tests were given in at least three copies. Continuous variables were expressed as the mean ± standard deviation (SD). Statistical analysis was performed using a one-way ANOVA to determine if there were significant differences between groups. *P* < 0.05 was considered statistically different. All statistical analyses were performed using GraphPad Prism-8 software.

### Ethics approval and consent to participate

This study was approved by the Animal Management and Ethics Committee of Gannan Medical University (NO. 2022151).

## Results

### CUR ameliorates gait abnormalities in rats caused by KOA

**Figure 2:**
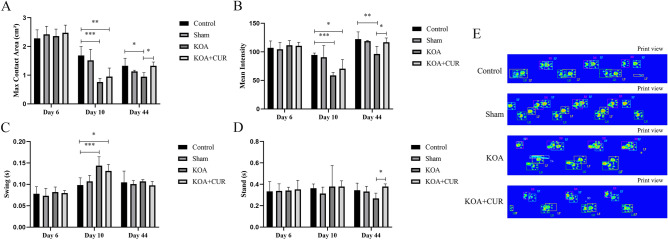
CUR ameliorates gait abnormalities in rats caused by KOA. (**A**) Effect of KOA on the maximum contact area of rats. Maximum contact area (cm^2^) represents the maximum surface area of the claw in contact with the glass plate. (**B**) Effect of KOA on the mean intensity of rats. Mean intensity represents the average intensity of the claw in contact with the glass plate. (**C**) Effect of KOA on the swing of rats. Swing (s) is the time that the claw did not make contact with the glass plate during a walking cycle. (**D**) Effect of KOA on the stand of rats. Stand (s) is the time that the claw is in contact with the glass plate. (**E**) This is the print view of the claw on the glass plate. Control represents the blank control rats. Sham represents sham-operated group rats. KOA represents the knee osteoarthritis model group rats. KOA + CUR represents the treated group rats with feeding CUR. *P < 0.05, **P < 0.01, ***P < 0.001. All data are expressed as mean ± SD, n = 8 for each group.

The gait parameters of rats were assessed using the CatWalk gait analysis system. In this test, we measured four gait parameters to show the pain-related behavior of KOA rats. As shown in Fig. 2, CatWalk gait analysis was performed 3 times, including one day before surgery, three days after surgery, and 35 days after intervention. The KOA group significantly reduced the maximum contact area (Fig. 2A) and mean intensity (Fig. 2B), and the swing duration (Fig. 2C) increased compared to the control group. This suggested that Hulth surgery induced abnormal movement patterns in rats. These conditions were significantly reversed by CUR treatment (150 mg/kg/day) in KOA rats, with an increase in maximum contact area, mean intensity and stand (Fig. 2D). These results suggest that CUR treatment attenuates pain-related behaviors. Printed views of the paws of each group of rats on the glass plate are shown in Fig. 2E.

### CUR improves KOA-induced histologic changes in knee joints

**Figure 3:**
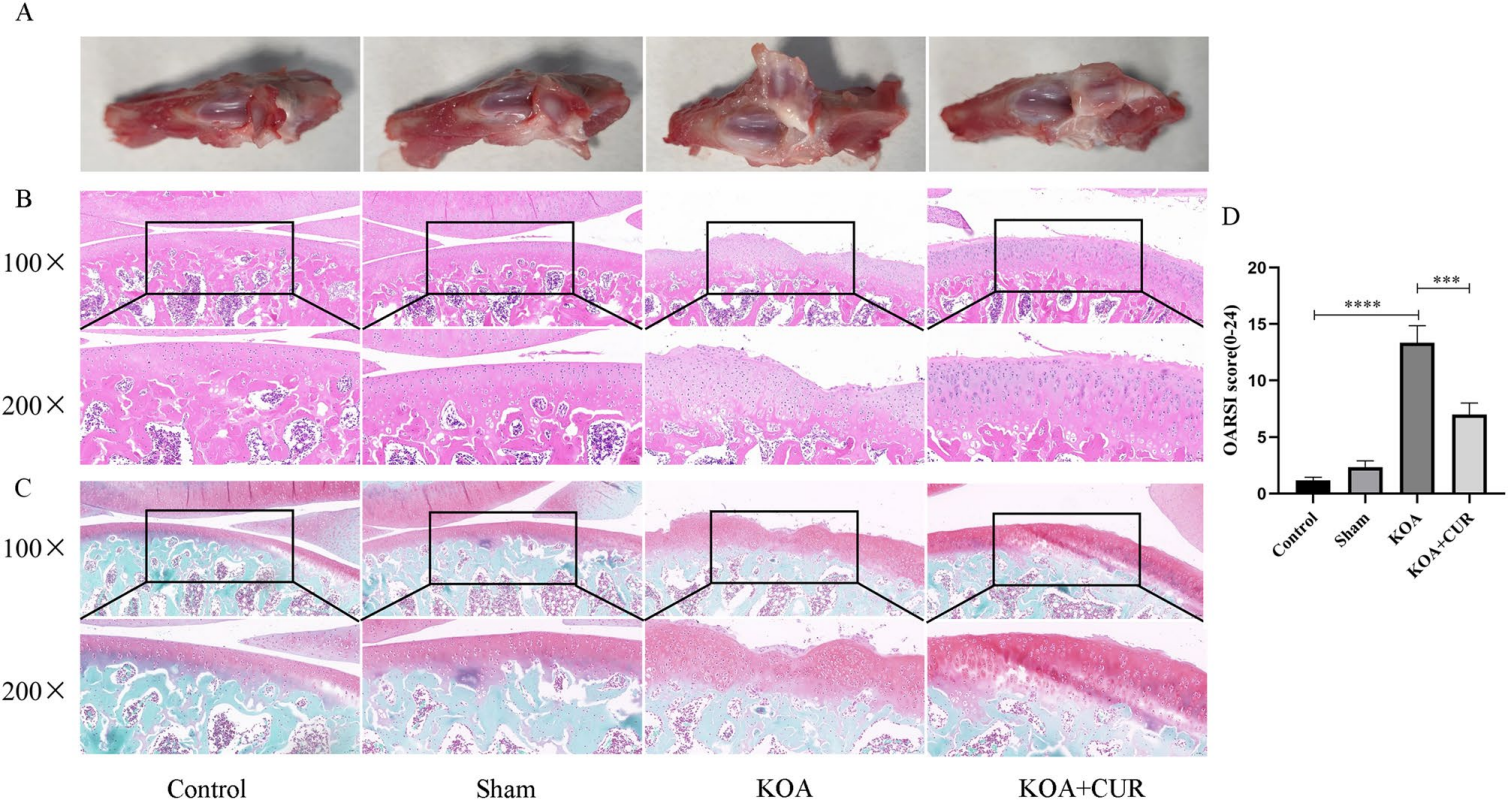
Cartilage changes in each group of rats. (**A**) Gross morphological images of the rat knee joints. (**B**) H&E images of rat knee joints. Images were acquired at 100 ×, scale bars = 100 μm. Images were acquired at 200 ×, scale bars = 50 μm. (**C**) Images of Safranin O/Fast Green staining of rat knee joints. Images were acquired at 100 ×, scale bars = 100 μm. Images were acquired at 200 × , scale bars = 50 μm. (**D**) OARSI scores of rats in each group. Control represents the blank control rats. Sham represents sham-operated group rats. KOA represents the knee osteoarthritis model group rats. KOA + CUR represents the treated group rats with feeding CUR. ***P < 0.001, ****P < 0.0001 All data are expressed as mean ± SD, n = 3 for each group.

KOA causes significant pathologic changes in the knee joint^65^. As shown in Fig. 3A, the morphology of the rat knee joint was observed, the cartilage surface of the rats in the KOA group was rough and dull, and defects were visible on the surface. The cartilage on the surface of the knee joint of the rats in the KOA + CUR group was smoother relative to that of the model group. As shown in Fig. 3B and C, we performed H&E staining and Safranin-O/Fast Green staining on the knee joints of rats in the KOA group. The staining results indicated that the cartilage surface layer of the control group rats was flatter and structurally intact, with a thicker cartilage layer and relatively more cells and proteoglycans. The joint surface of the KOA group rats was rough, with obvious cracks, a thinner cartilage layer, and fewer cells visible in irregular tidal lines. Compared with KOA group rats, the cartilage surface of KOA + CUR group rats was slightly flat, without cracks, and the cartilage layer was thicker with more cells. As shown in Fig. 3D, We used the OARSI scoring method to determine the severity of osteoarthritis^42^. We found that rats in the KOA group scored considerably higher (*P* < 0.0001) compared to rats in the control group. The severity of cartilage damage in the KOA + CUR group rats was significantly lower than that in the KOA group of rats, and the difference between the two groups was statistically significant (*P* < 0.001).

### CUR can improve quadriceps femoris muscle atrophy due to KOA

**Figure 4:**
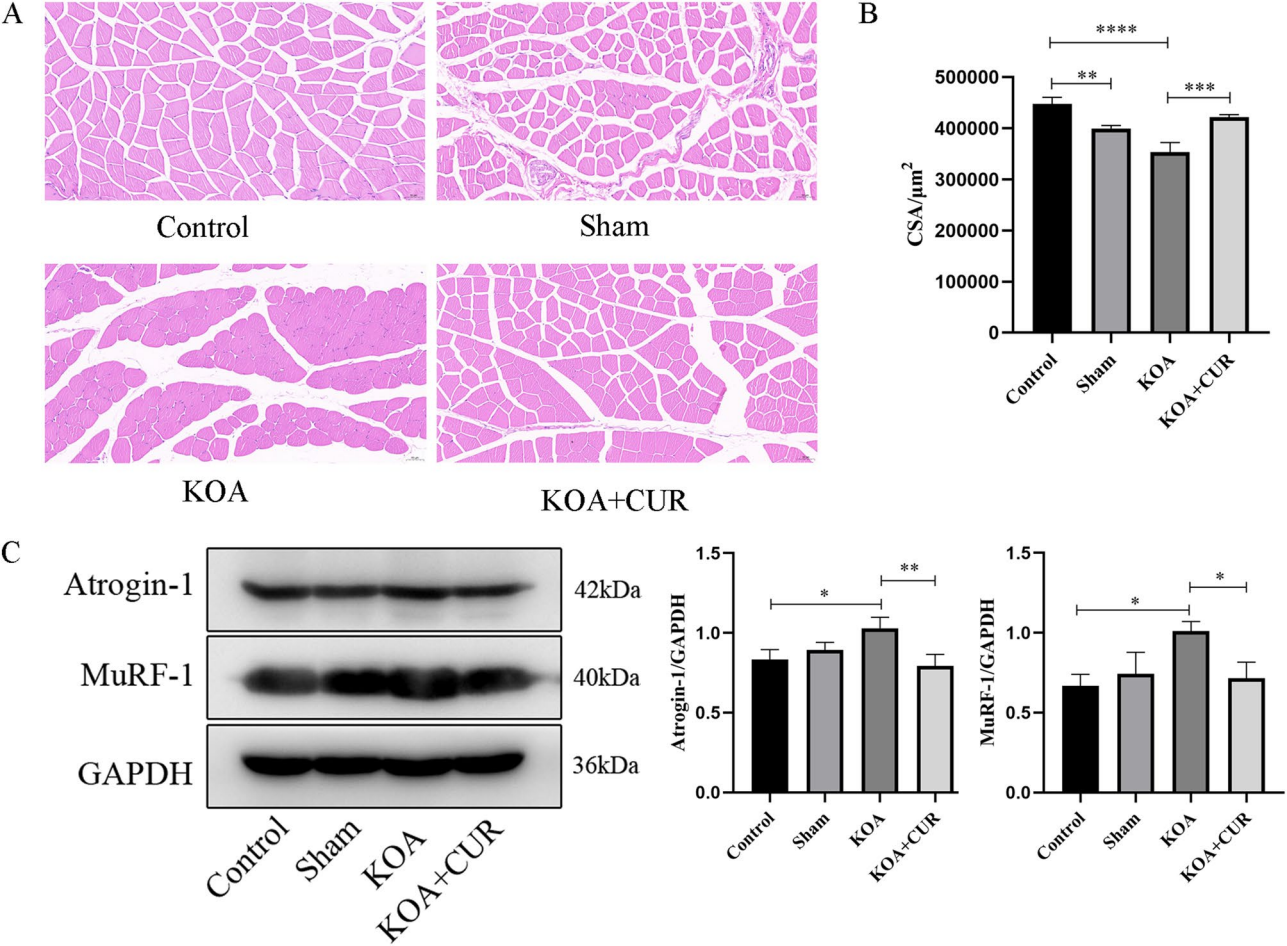
CUR can improve quadriceps femoris muscle atrophy due to KOA. (**A**) Representative H&E staining images of the quadriceps femoris muscle. Scale bar = 50 µm. (**B**) Myofiber CSA determined from cross-sections of the quadriceps femoris muscle. (**C**) Changes in MuRF-1 and Atrogin-1 expression were assessed by Western blotting. Equal protein loading was normalized by GAPDH. Original blots/gels were presented in Supplementary Figs. S1–S3. Control represents the blank control rats. Sham represents sham-operated group rats. KOA represents the knee osteoarthritis model group rats. KOA + CUR represents the treated group rats with feeding CUR. *P < 0.05, **P < 0.01, ***P < 0.001, **** *P* < 0.0001. All data are expressed as mean ± SD, n = 3 for each group.

As shown in Fig. 4A, the quadriceps femoris muscle of the rats in the KOA group showed atrophy and the morphology of the myocytes became more rounded. We calculated the CSA of the quadriceps femoris muscle in each group by the results of H&E staining and found that the CSA of the quadriceps femoris muscle in the KOA group was a lot smaller than that in the control group, and the CSA of the rats in the KOA + CUR group was more than that in the KOA group (Fig. 4B). Atrogin-1 and MuRF-1 are considered markers associated with skeletal muscle atrophy^66^. As shown in Fig. 4C, compared with the control group, the expression of Atrogin-1 and MuRF-1 in the quadriceps femoris muscle of the KOA group was significantly higher, while compared with the KOA group, the expression of Atrogin-1 and MuRF-1 in the quadriceps femoris muscle of the KOA + CUR group was significantly decreased. The above results indicate that KOA caused quadriceps femoris muscle atrophy, and this atrophy improved after CUR treatment.

### Autophagy is hyperactivated in the quadriceps femoris muscle of KOA rats

**Figure 5:**
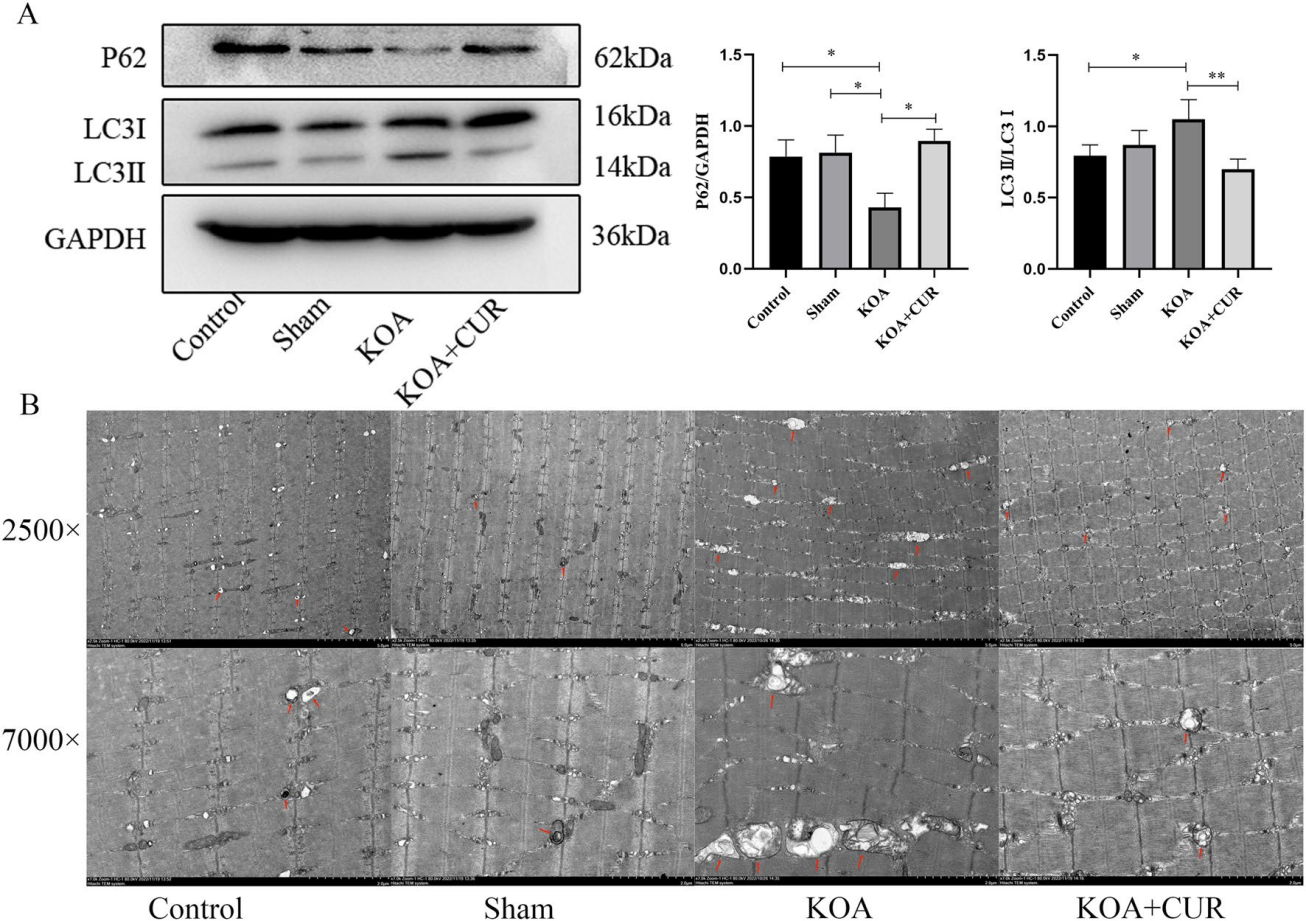
Autophagy is hyperactivated in the quadriceps femoris muscle of KOA rats. (**A**) Changes in LC3 and P62 expression were assessed by Western blotting. Equal protein loading was normalized by GAPDH. Original blots/gels were presented in Supplementary Figs. S4–S6. *P < 0.05, **P < 0.01, ***P < 0.001. All data are expressed as mean ± SD, n = 3 for each group. (**B**) Abnormal morphology of autophagosomes and mitochondria in the quadriceps femoris muscle of KOA rats. Autophagosomes (red arrows). Images were acquired at 2500 × , scale bars = 5 μm. Images were acquired at 7000 × , scale bars = 2 μm. Control represents the blank control rats. Sham represents sham-operated group rats. KOA represents the KOA model group rats. KOA + CUR represents the treated group rats with feeding CUR.

We observed the changes in autophagy within the quadriceps femoris muscle by western blotting analysis and transmission electron microscopy. Basal levels of autophagy are protective of cells, but over-activation of autophagy leads to cell death. P62/SQSTM1 (hereafter as p62), a major defense protein, regulates autophagy of mitochondrial debris post-oxidative stress. P62 form autophagosomes by binding to light chain 3 (LC3) on the mitochondrial membrane, which degrade into autolysosomes, thus triggering autophagic flu^67^. As shown in the Fig. 5A, compared with control rats, autophagy was overactivated in the quadriceps femoris muscle of the KOA group, whereas autophagy was inhibited in the quadriceps femoris muscle of the KOA + CUR group. As shown in the Fig. 5B, the quadriceps femoris muscle of control and sham group rats showed the ultrastructure of normal muscle, normal mitochondrial morphology, and the presence of a small number of autophagosomes (red arrows). However, in the KOA group, it showed more autophagosomes (red arrows), enlarged and fragmented mitochondria, and disorganized cristae. There was a decrease in autophagosomes and an improvement in mitochondrial morphology within the quadriceps muscle of rats in the CUR + KOA group compared to the KOA group.

### CUR alleviates oxidative stress and inhibits autophagy via SIRT3-SOD2

**Figure 6:**
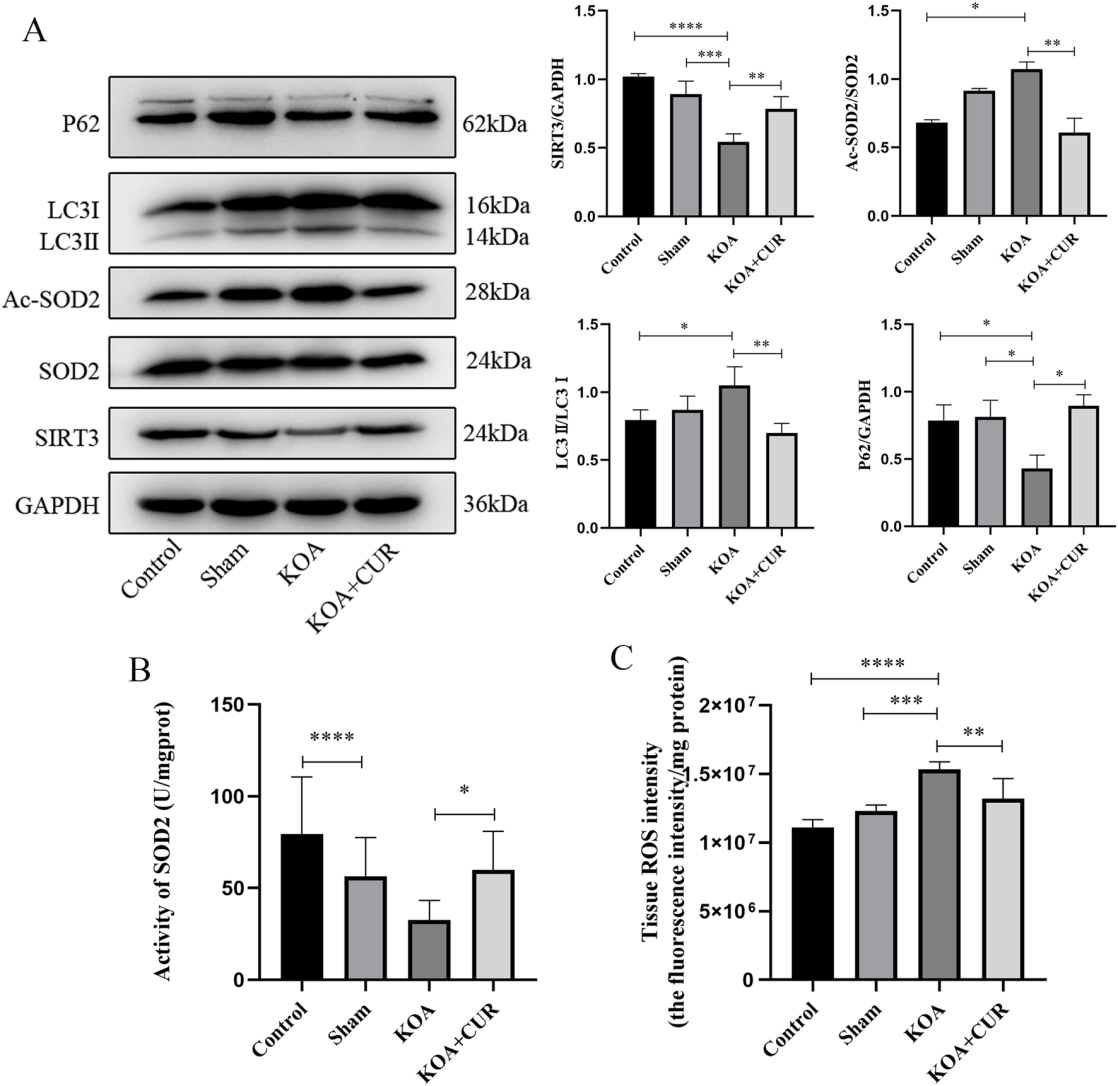
CUR alleviates oxidative stress and inhibits autophagy via SIRT3-SOD2. (**A**) Protein expressions of SIRT3, SOD2, Ac-SOD2, LC3 and P62 was detected by western blotting. Equal protein loading was normalized by GAPDH. Original blots/gels were presented in Supplementary Figs. S7–S12. (**B**) ROS content in the quadriceps femoris muscle of rats in each group. (**C**) Activity of SOD2 in quadriceps femoris muscle of rats in each group. Control represents the blank control rats. Sham represents sham-operated group rats. KOA represents the knee osteoarthritis model group rats. KOA + CUR represents the treated group rats with feeding CUR. *P < 0.05, **P < 0.01, ***P < 0.001, ****P < 0.0001. All data are expressed as mean ± SD, n = 3 for each group.

Excessive accumulation of ROS can over-activate autophagy^68^. The main scavenger of oxygen radicals in mitochondria is SOD2^69^ and its activity is mostly modulated by the deacetylase SIRT3 in mitochondria^70,71^. As shown in the Fig. 6A, compared with the control group, the expression of deacetylated SOD2 was decreased, the activity of SOD2 was decreased (Fig. 6B), ROS accumulation was increased (Fig. 6C), and autophagy was over-activated in the quadriceps femoris of rats in the KOA group. The expression of SIRT3 in the quadriceps femoris muscle of rats after CUR treatment was increased, and deacetylation acted on SOD2, which enhanced SOD2 activity and scavenged ROS, thus alleviating the oxidative stress response. Autophagy induced by ROS was inhibited and quadriceps femoris muscle atrophy was improved.

## Discussion

Quadriceps femoris muscle weakness and atrophy are common complications in patients with KOA^3,4,72^. It is not only the active muscle that produces knee extension movements, but also provides knee stability^6^. Weakness and atrophy of the quadriceps femoris muscle can further affect the onset and progression of KOA^73–76^. Some studies have shown that exercise training to strengthen the quadriceps femoris muscle can be used to treat KOA^11,77^. In our study, we found that the articular cartilage of the rats in the KOA group was severely damaged, and the quadriceps femoris muscle was markedly atrophied. In the CUR-treated group, the atrophy of the quadriceps femoris muscle was markedly ameliorated, and the damage of the knee joint cartilage was more KOA group rats also improved, which may be achieved by regulating the SIRT3-SOD2 signaling pathway. It has also been previously shown that CUR can improve muscle atrophy by modulating SIRT3 activity^64,78^.

Muscle atrophy occurs because of an imbalance in protein synthesis and degradation. During skeletal muscle atrophy, two major protein hydrolysis systems are activated: the ubiquitin–proteasome system and the autophagy-lysosome system^12^. Autophagy helps maintain muscle mass. When undergoing fasting or denervation stimulation autophagy is inhibited and severe muscle atrophy occurs^13^. However, over-activated autophagy can exacerbate cellular damage by engulfing proteins, mitochondria, or other organelles critical to cellular health^79^. How autophagy changes in KOA-induced quadriceps femoris muscle atrophy has not been reported. In our study, autophagy-associated proteins were determined by Western blotting and the presence of autophagic vesicles was observed by transmission electron microscopy. The results showed that LC3II/LC3I was significantly increased and P62 expression was decreased in the quadriceps femoris muscle of rats in the KOA group. Autophagic vesicles were significantly increased in the quadriceps femoris muscle of rats in the KOA group, as observed by transmission electron microscopy. This indicated that autophagy was activated in the atrophied quadriceps femoris muscle of KOA rats. Our findings are in agreement with Zhang, Y. Y, et al. that autophagy is hyperactivated in rectus abdominis atrophy induced by chronic kidney disease^64^. Yang, X. et al. also found that the gastrocnemius and tibialis anterior muscles underwent rapid atrophy after denervation and that mitochondrial autophagic activity was significantly enhanced^80^. It has been shown that ROS levels rise when muscle atrophy occurs^16^. It has been shown that ROS can activate the ubiquitin proteasome system to degrade Bcl-2, leading to increased Beclin-1 expression and the induction of autophagy^18,81,82^. We examined the changes in ROS content in the quadriceps femoris muscle of KOA rats. The results showed that ROS were significantly increased in the quadriceps femoris muscle of rats in the KOA group compared with the control group. Our findings suggested that quadriceps femoris muscle atrophy in KOA rats may be due to excessive activation of autophagy by increased ROS levels. It has also been previously shown that mitochondrial autophagy activity is enhanced and ROS generation is increased in disused muscle atrophy^17^.

SOD2 is the main scavenger of free radicals in mitochondria^83,84^. SOD2 activity is important for maintaining intracellular oxygen radical homeostasis^85,86^ and its activity is regulated by the deacetylase SIRT3^85–87^. SIRT3 is the most powerful mitochondrial deacetylase and is directly engaged in the regulation of mitochondrial energy synthesis and oxygen radical levels^88^. Acting on the SIRT3-SOD2 pathway alleviates ischemia/reperfusion-induced myocardial oxidative stress and apoptosis^89^. Hypertrophic preconditioning attenuates myocardial ischemia–reperfusion injury by modulating SIRT3-SOD2 dependent autophagy^90^. In our study, we found that the expression of SIRT3 was decreased, the acetylation level of SOD2 was increased, SOD2 activity was decreased, ROS content was increased, and autophagy was activated in the atrophied quadriceps femoris muscle of rats in the KOA group. From these results, it can be hypothesized that SIRT3-SOD2-mediated autophagy may be an important mechanism of quadriceps femoris muscle atrophy in KOA rats.

CUR is a natural antioxidant derived from the roots of ginger, a plant in the ginger family^22^. It is considered an activator of SIRT3^28,91,92^. CUR exerts a protective effect on bone marrow by reducing ROS-stimulated autophagic cell death in a SIRT3-SOD2 pathway-dependent manner^69^. CUR attenuates ischemia–reperfusion-induced myocardial injury by activating SIRT3^78^. KOA rats were treated with CUR for 35 days. Our results showed that SIRT3 expression was increased, SOD2 acetylation level was decreased, and SOD2 activity was increased in the quadriceps femoris muscle of KOA rats, which resulted in the scavenging of ROS and the inhibition of autophagy. The expression of Atrogin-1 and MuRF-1 in the quadriceps femoris muscle of KOA rats was significantly decreased and CSA was significantly increased. From the above results, we hypothesized that CUR improves quadriceps femoris muscle atrophy in KOA rats by activating the SIRT3-SOD2 pathway and scavenging ROS to inhibit over-activated autophagy.

Our study had some limitations. We did not use inhibitors to clarify that CUR acts on the SIRT3-SOD2 pathway, and can only say that CUR may act through the SIRT3-SOD2 pathway to scavenge ROS, inhibit over-activated autophagy, improve quadriceps femoris atrophy, and further improve the KOA process.

## Conclusion

In this study, we propose for the first time a possible mechanism by which KOA induces quadriceps femoris muscle atrophy through ROS-dependent autophagy. CUR was applied to promote quadriceps femoris muscle regeneration in KOA rats. It may play a protective role in vivo by clearing ROS through SIRT3-SOD2 pathway to inhibit autophagy. These findings provide a new idea for the treatment of KOA-induced quadriceps femoris muscle atrophy and a new strategy for the treatment of KOA.

### Supplementary Information

Supplementary Figures.

## Data Availability

The data presented in this study are available on request from the corresponding author.

## Abbreviations

KOA: Knee osteoarthritis
ROS: Reactive oxygen species
SIRT3: Sirutin3
SOD2: Superoxide dismutase 2
CUR: Curcumin
OARSI: Osteoarthritis Research Society International

## References

[1] Vos T (2012). Years lived with disability (YLDs) for 1160 sequelae of 289 diseases and injuries 1990–2010: A systematic analysis for the Global Burden of Disease Study 2010. Lancet (Lond. Engl.).

[2] Zhang L (2022). Relationship between classification of fabellae and the severity of knee osteoarthritis: A relevant study in the chinese population. Orthop. Surg..

[3] Mohajer B (2022). Role of thigh muscle changes in knee osteoarthritis outcomes: Osteoarthritis initiative data. Radiology.

[4] Cunha JE (2019). Knee osteoarthritis induces atrophy and neuromuscular junction remodeling in the quadriceps and tibialis anterior muscles of rats. Sci. Rep..

[5] Terracciano C (2013). Differential features of muscle fiber atrophy in osteoporosis and osteoarthritis. Osteoporos. Int. J. Estab. Result Cooper. Eur. Found. Osteoporos. Natl. Osteoporos. Found. USA.

[6] Bennell KL, Wrigley TV, Hunt MA, Lim BW, Hinman RS (2013). Update on the role of muscle in the genesis and management of knee osteoarthritis. Rheum. Dis. Clin. N. Am..

[7] Hurley MV, Newham DJ (1993). The influence of arthrogenous muscle inhibition on quadriceps rehabilitation of patients with early, unilateral osteoarthritic knees. Br. J. Rheumatol..

[8] Kittelson AJ, Thomas AC, Kluger BM, Stevens-Lapsley JE (2014). Corticospinal and intracortical excitability of the quadriceps in patients with knee osteoarthritis. Exp. Brain Res..

[9] Slemenda C (1997). Quadriceps weakness and osteoarthritis of the knee. Ann. Intern. Med..

[10] Roos EM, Herzog W, Block JA, Bennell KL (2011). Muscle weakness, afferent sensory dysfunction and exercise in knee osteoarthritis. Nat. Rev. Rheumatol..

[11] Kus G, Yeldan I (2019). Strengthening the quadriceps femoris muscle versus other knee training programs for the treatment of knee osteoarthritis. Rheumatol. Int..

[12] Milan G (2015). Regulation of autophagy and the ubiquitin-proteasome system by the FoxO transcriptional network during muscle atrophy. Nat. Commun..

[13] Masiero E (2009). Autophagy is required to maintain muscle mass. Cell Metab..

[14] Masiero E, Sandri M (2010). Autophagy inhibition induces atrophy and myopathy in adult skeletal muscles. Autophagy.

[15] Dobrowolny G (2008). Skeletal muscle is a primary target of SOD1G93A-mediated toxicity. Cell Metab..

[16] Muller FL (2007). Denervation-induced skeletal muscle atrophy is associated with increased mitochondrial ROS production. Am. J. Physiol. Regul. Integr. Comp. Physiol..

[17] Yamashita SI (2021). Mitophagy reporter mouse analysis reveals increased mitophagy activity in disuse-induced muscle atrophy. J. Cell. Physiol..

[18] Zheng Y, Shi B, Ma M, Wu X, Lin X (2019). The novel relationship between Sirt3 and autophagy in myocardial ischemia-reperfusion. J. Cell. Physiol..

[19] Bause AS, Haigis MC (2013). SIRT3 regulation of mitochondrial oxidative stress. Exp. Gerontol..

[20] Qiu X, Brown K, Hirschey MD, Verdin E, Chen D (2010). Calorie restriction reduces oxidative stress by SIRT3-mediated SOD2 activation. Cell Metab..

[21] Liang Q (2013). Bioenergetic and autophagic control by Sirt3 in response to nutrient deprivation in mouse embryonic fibroblasts. Biochem. J..

[22] Zia A, Farkhondeh T, Pourbagher-Shahri AM, Samarghandian S (2021). The role of curcumin in aging and senescence: Molecular mechanisms. Biomed. Pharmacother. Biomed. Pharmacother..

[23] Zhang J (2014). Assessment of free radicals scavenging activity of seven natural pigments and protective effects in AAPH-challenged chicken erythrocytes. Food Chem..

[24] Campbell MS, Carlini NA, Fleenor BS (2021). Influence of curcumin on performance and post-exercise recovery. Crit. Rev. Food Sci. Nutr..

[25] Fernández-Lázaro D (2020). Modulation of exercise-induced muscle damage, inflammation, and oxidative markers by curcumin supplementation in a physically active population: A systematic review. Nutrients.

[26] Nanavati K, Rutherfurd-Markwick K, Lee SJ, Bishop NC, Ali A (2022). Effect of curcumin supplementation on exercise-induced muscle damage: A narrative review. Eur. J Nutr..

[27] Dias KA (2022). Curcumin-added whey protein positively modulates skeletal muscle inflammation and oxidative damage after exhaustive exercise. Nutrients.

[28] Zhang M (2017). Curcumin attenuates skeletal muscle mitochondrial impairment in COPD rats: PGC-1α/SIRT3 pathway involved. Chem.-Biol. Interact..

[29] Sadeghi A, Rostamirad A, Seyyedebrahimi S, Meshkani R (2018). Curcumin ameliorates palmitate-induced inflammation in skeletal muscle cells by regulating JNK/NF-kB pathway and ROS production. Inflammopharmacology.

[30] Yu T (2022). Protective effects of dietary curcumin and astaxanthin against heat-induced ROS production and skeletal muscle injury in male and female C57BL/6J mice. Life Sci..

[31] Mañas-García L, Bargalló N, Gea J, Barreiro E (2020). Muscle phenotype, proteolysis, and atrophy signaling during reloading in mice: Effects of curcumin on the gastrocnemius. Nutrients.

[32] Mañas-García L, Guitart M, Duran X, Barreiro E (2020). Satellite cells and markers of muscle regeneration during unloading and reloading: Effects of treatment with resveratrol and curcumin. Nutrients.

[33] Lee DY (2021). Curcumin attenuates sarcopenia in chronic forced exercise executed aged mice by regulating muscle degradation and protein synthesis with antioxidant and anti-inflammatory effects. J. Agric. Food Chem..

[34] Saud Gany SL, Chin KY, Tan JK, Aminuddin A, Makpol S (2023). Curcumin as a therapeutic agent for sarcopenia. Nutrients.

[35] Receno CN (2019). Effects of prolonged dietary curcumin exposure on skeletal muscle biochemical and functional responses of aged male rats. Int. J. Mol. Sci..

[36] Zhao J (2024). Efficacy and safety of curcumin therapy for knee osteoarthritis: A Bayesian network meta-analysis. J. Ethnopharmacol..

[37] Hsiao AF (2021). The efficacy of high- and low-dose curcumin in knee osteoarthritis: A systematic review and meta-analysis. Complement. Ther. Med..

[38] Wang Z (2021). Efficacy and safety of turmeric extracts for the treatment of knee osteoarthritis: A systematic review and meta-analysis of randomised controlled trials. Curr. Rheumatol. Rep..

[39] Guan T (2022). Combined administration of curcumin and chondroitin sulfate alleviates cartilage injury and inflammation via NF-κB pathway in knee osteoarthritis rats. Front. Pharmacol..

[40] Zhang Y, Zeng Y (2019). Curcumin reduces inflammation in knee osteoarthritis rats through blocking TLR4 /MyD88/NF-κB signal pathway. Drug Dev. Res..

[41] Chen B (2023). Combination of curcumin and catalase protects against chondrocyte injury and knee osteoarthritis progression by suppressing oxidative stress. Biomed. Pharmacother. Biomed. Pharmacother.

[42] Jin Z (2022). Curcumin exerts chondroprotective effects against osteoarthritis by promoting AMPK/PINK1/Parkin-mediated mitophagy. Biomed. Pharmacother. Biomed. Pharmacother..

[43] Hu Z (2021). Differentially expressed genes accompanying neurobehavioral deficits in a modified rat model of vascular dementia. Neurosci. Lett..

[44] Liu F, Yang H, Li D, Wu X, Han Q (2021). Punicalagin attenuates osteoarthritis progression via regulating Foxo1/Prg4/HIF3α axis. Bone.

[45] Timotius IK (2023). CatWalk XT gait parameters: A review of reported parameters in pre-clinical studies of multiple central nervous system and peripheral nervous system disease models. Front. Behav. Neurosci..

[46] Ren J (2023). Schwann cell-derived exosomes containing MFG-E8 modify macrophage/microglial polarization for attenuating inflammation via the SOCS3/STAT3 pathway after spinal cord injury. Cell Death Dis..

[47] Sun Y, Jia D, Xue M, Huang Z, Huang C (2022). Trifluoro-icaritin alleviates chronic inflammatory pain through α7nAChR-mediated suppression of HMGB1/NF-κB signaling in the spinal cord of rats. Brain Res. Bull..

[48] Zhuang H, Ren X, Jiang F, Zhou P (2023). Indole-3-propionic acid alleviates chondrocytes inflammation and osteoarthritis via the AhR/NF-κB axis. Mol. Med..

[49] Zheng X (2023). Paroxetine attenuates chondrocyte pyroptosis and inhibits osteoclast formation by inhibiting NF-κB pathway activation to delay osteoarthritis progression. Drug Des. Dev. Ther..

[50] Chien SY (2020). Noggin inhibits IL-1β and BMP-2 expression, and attenuates cartilage degeneration and subchondral bone destruction in experimental osteoarthritis. Cells.

[51] Liu SC (2017). Soya-cerebroside, an extract of *Cordyceps*
*militaris*, suppresses monocyte migration and prevents cartilage degradation in inflammatory animal models. Sci. Rep..

[52] Lin CY (2017). Brain-derived neurotrophic factor promotes VEGF-C-dependent lymphangiogenesis by suppressing miR-624-3p in human chondrosarcoma cells. Cell Death Dis..

[53] Ono T, Takada S, Kinugawa S, Tsutsui H (2015). Curcumin ameliorates skeletal muscle atrophy in type 1 diabetic mice by inhibiting protein ubiquitination. Exp. Physiol..

[54] Gao P, Wu B, Ding Y, Yin B, Gu H (2023). circEXOC5 promotes acute lung injury through the PTBP1/Skp2/Runx2 axis to activate autophagy. Life Sci. Alliance.

[55] Zhou Y (2023). A novel long noncoding RNA SP100-AS1 induces radioresistance of colorectal cancer via sponging miR-622 and stabilizing ATG3. Cell Death Differ..

[56] Dindi UMR (2023). In-silico and in-vitro functional validation of imidazole derivatives as potential sirtuin inhibitor. Front. Med..

[57] Xie Z (2023). Healthy human fecal microbiota transplantation into mice attenuates mptp-induced neurotoxicity via AMPK/SOD2 pathway. Aging Dis..

[58] Huang YM (2023). Glucagon-like peptide-2 ameliorates age-associated bone loss and gut barrier dysfunction in senescence-accelerated mouse prone 6 mice. Gerontology.

[59] Zhou R (2022). A signalling pathway for transcriptional regulation of sleep amount in mice. Nature.

[60] Pi H (2015). SIRT3-SOD2-mROS-dependent autophagy in cadmium-induced hepatotoxicity and salvage by melatonin. Autophagy.

[61] Zhong G (2019). Dopamine-melanin nanoparticles scavenge reactive oxygen and nitrogen species and activate autophagy for osteoarthritis therapy. Nanoscale.

[62] Lu Z (2019). An injectable collagen-genipin-carbon dot hydrogel combined with photodynamic therapy to enhance chondrogenesis. Biomaterials.

[63] Wei L (2015). Oroxylin A inhibits glycolysis-dependent proliferation of human breast cancer via promoting SIRT3-mediated SOD2 transcription and HIF1α destabilization. Cell Death Dis..

[64] Zhang YY (2019). CKD autophagy activation and skeletal muscle atrophy-a preliminary study of mitophagy and inflammation. Eur. J. Clin. Nutr..

[65] Cheng JH (2022). Pathological, morphometric and correlation analysis of the modified mankin score, tidemark roughness and calcified cartilage thickness in rat knee osteoarthritis after extracorporeal shockwave therapy. Int. J. Med. Sci..

[66] Shang GK (2020). Sarcopenia is attenuated by TRB3 knockout in aging mice via the alleviation of atrophy and fibrosis of skeletal muscles. J. Cachexia Sarcopenia Muscle.

[67] Zeng X (2022). Activated Drp1 regulates p62-mediated autophagic flux and aggravates inflammation in cerebral ischemia-reperfusion via the ROS-RIP1/RIP3-exosome axis. Milit. Med. Res..

[68] Sena LA, Chandel NS (2012). Physiological roles of mitochondrial reactive oxygen species. Mol. Cell.

[69] Zhou S (2021). Iron overload adversely effects bone marrow haematogenesis via SIRT-SOD2-mROS in a process ameliorated by curcumin. Cell. Mol. Biol. Lett..

[70] Angelucci E (2014). Deferasirox for transfusion-dependent patients with myelodysplastic syndromes: Safety, efficacy, and beyond (GIMEMA MDS0306 Trial). Eur. J. Haematol..

[71] Di Tucci AA (2007). Correction of anemia in a transfusion-dependent patient with primary myelofibrosis receiving iron chelation therapy with deferasirox (Exjade, ICL670). Eur. J. Haematol..

[72] Noehren B (2018). Alterations in quadriceps muscle cellular and molecular properties in adults with moderate knee osteoarthritis. Osteoarthr. Cartil..

[73] Øiestad BE, Juhl CB, Culvenor AG, Berg B, Thorlund JB (2022). Knee extensor muscle weakness is a risk factor for the development of knee osteoarthritis: An updated systematic review and meta-analysis including 46 819 men and women. Br. J. Sports Med..

[74] Dell'isola A, Wirth W, Steultjens M, Eckstein F, Culvenor AG (2018). Knee extensor muscle weakness and radiographic knee osteoarthritis progression. Acta Orthop..

[75] Segal NA, Glass NA (2011). Is quadriceps muscle weakness a risk factor for incident or progressive knee osteoarthritis?. Physician Sportsmed..

[76] Takagi S (2018). Quadriceps muscle weakness is related to increased risk of radiographic knee OA but not its progression in both women and men: The Matsudai Knee Osteoarthritis Survey. Knee Surg. Sports Traumatol. Arthrosc. Off. J. ESSKA.

[77] Xie Y (2018). Quadriceps combined with hip abductor strengthening versus quadriceps strengthening in treating knee osteoarthritis: A study protocol for a randomized controlled trial. BMC Musculoskelet. Disord..

[78] Wang R (2018). Curcumin attenuates IR-induced myocardial injury by activating SIRT3. Eur. Rev. Med. Pharmacol. Sci..

[79] Mizushima N, Komatsu M (2011). Autophagy: Renovation of cells and tissues. Cell.

[80] Yang X (2020). Denervation drives skeletal muscle atrophy and induces mitochondrial dysfunction, mitophagy and apoptosis via miR-142a-5p/MFN1 axis. Theranostics.

[81] Breitschopf K, Haendeler J, Malchow P, Zeiher AM, Dimmeler S (2000). Posttranslational modification of Bcl-2 facilitates its proteasome-dependent degradation: molecular characterization of the involved signaling pathway. Mol. Cell. Biol..

[82] Pattingre S (2005). Bcl-2 antiapoptotic proteins inhibit Beclin 1-dependent autophagy. Cell.

[83] Zeng L (2014). Age-related decrease in the mitochondrial sirtuin deacetylase Sirt3 expression associated with ROS accumulation in the auditory cortex of the mimetic aging rat model. PLoS One.

[84] Li M, Chiu JF, Mossman BT, Fukagawa NK (2006). Down-regulation of manganese-superoxide dismutase through phosphorylation of FOXO3a by Akt in explanted vascular smooth muscle cells from old rats. J. Biol. Chem..

[85] Chen Y (2011). Tumour suppressor SIRT3 deacetylates and activates manganese superoxide dismutase to scavenge ROS. EMBO Rep..

[86] Zhu Y (2012). Exploring the electrostatic repulsion model in the role of Sirt3 in directing MnSOD acetylation status and enzymatic activity. Free Radic. Biol. Med..

[87] Tao R (2010). Sirt3-mediated deacetylation of evolutionarily conserved lysine 122 regulates MnSOD activity in response to stress. Mol. Cell.

[88] Giralt A, Villarroya F (2012). SIRT3, a pivotal actor in mitochondrial functions: Metabolism, cell death and aging. Biochem. J..

[89] Chang G, Chen Y, Zhang H, Zhou W (2019). Trans sodium crocetinate alleviates ischemia/reperfusion-induced myocardial oxidative stress and apoptosis via the SIRT3/FOXO3a/SOD2 signaling pathway. Int. Immunopharmacol..

[90] Ma LL (2021). Hypertrophic preconditioning attenuates myocardial ischaemia-reperfusion injury by modulating SIRT3-SOD2-mROS-dependent autophagy. Cell Prolif..

[91] Ungurianu A, Zanfirescu A, Margină D (2022). Regulation of gene expression through food-curcumin as a sirtuin activity modulator. Plants.

[92] Wiciński M (2023). Natural phytochemicals as SIRT activators-focus on potential biochemical mechanisms. Nutrients.

