# Supplementary material for: Curcumin regulates autophagy through SIRT3-SOD2-ROS signaling pathway to improve quadriceps femoris muscle atrophy in KOA rat model
Source: Sci Rep. 2024 Apr 8;14:8176. doi: 10.1038/s41598-024-58375-2 (PMC11001965; doi:10.1038/s41598-024-58375-2)

Figure S1


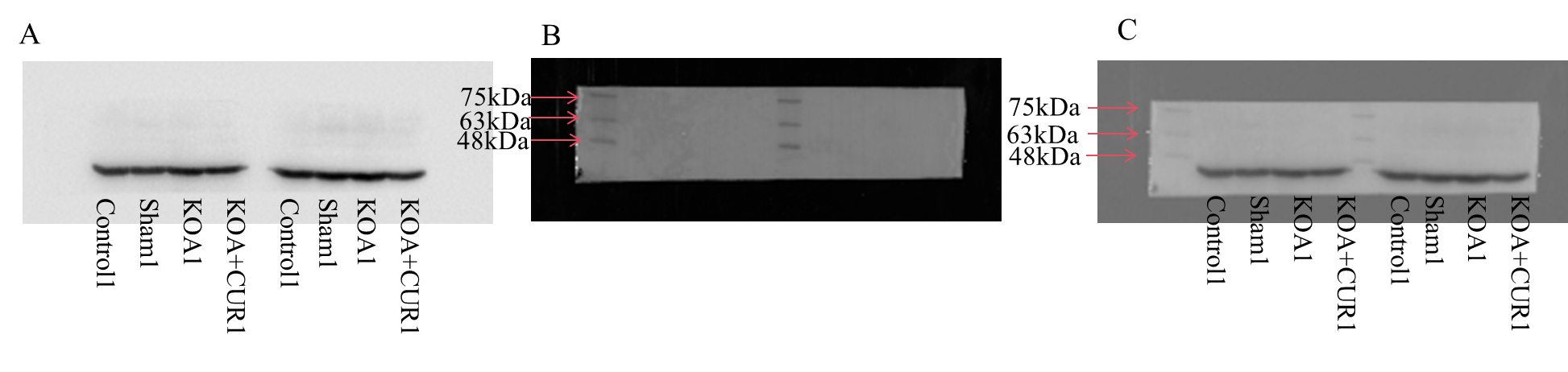


Original blot images of Atrogin-1. The left side of the blot (A) we used in Figure 4.

(A) Blot image of Atrogin-1.

(B) Protein marker image of Atrogin-1.

(C) Blot image of Atrogin-1 (A) merged with protein marker image (B).

Figure S2


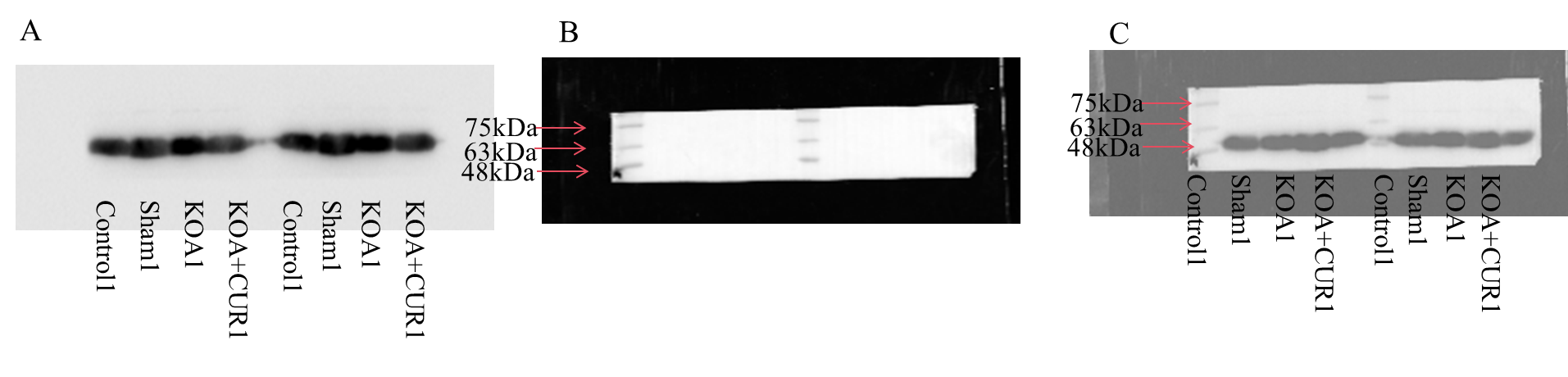


Original blot images of MuRF-1. The right side of the blot (A) we used in Figure 4.

(A) Blot image of MuRF-1.

(B) Protein marker image of MuRF-1.

(C) Blot image of MuRF-1 (A) merged with protein marker image (B).

Figure S3


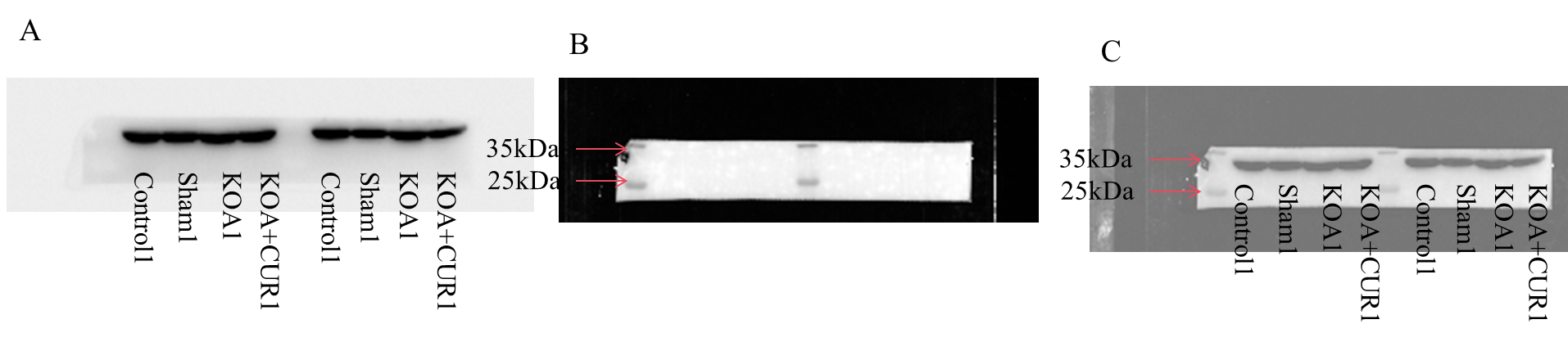


Original blot images of GAPDH. The left side of the blot (A) we used in Figure 4.

(A) Blot image of GAPDH.

(B) Protein marker image of GAPDH.

(C) Blot image of GAPDH (A) merged with protein marker image (B).

Figure S4


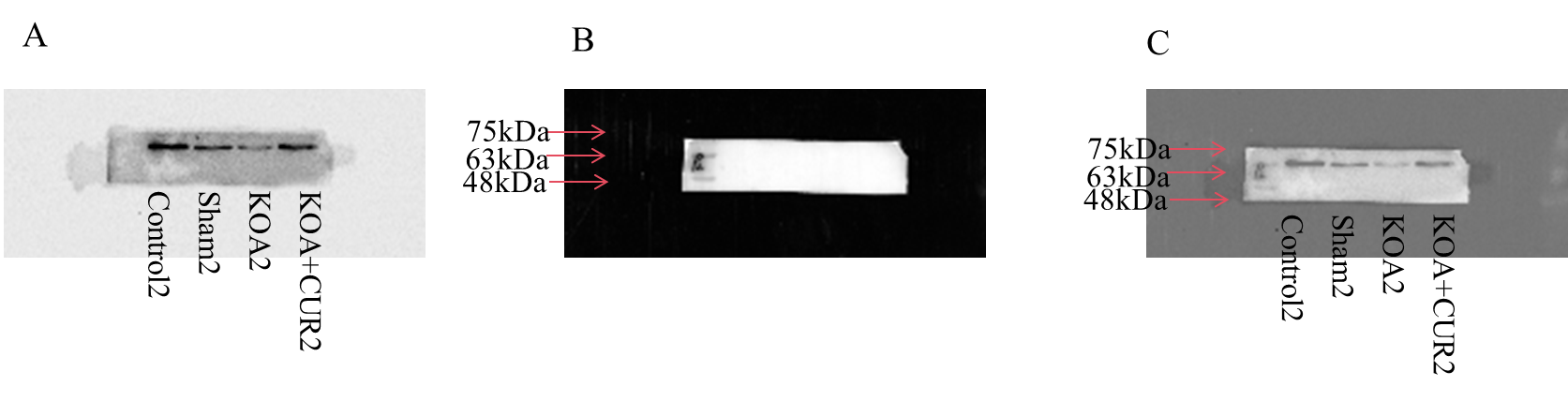


Original blot images of P62. The blot (A) we used in Figure 5.

(A) Blot image of P62.

(B) Protein marker image of P62.

(C) Blot image of P62 (A) merged with protein marker image (B).

Figure S5


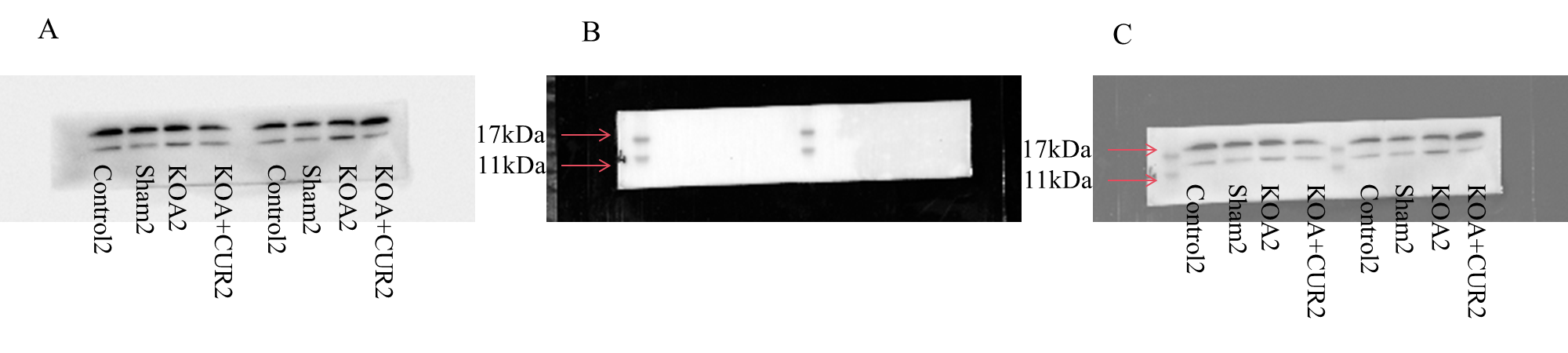


Original blot images of LC3. The right side of the blot (A) we used in Figure 5.

(A) Blot image of LC3.

(B) Protein marker image of LC3.

(C) Blot image of LC3 (A) merged with protein marker image (B).

Figure S6


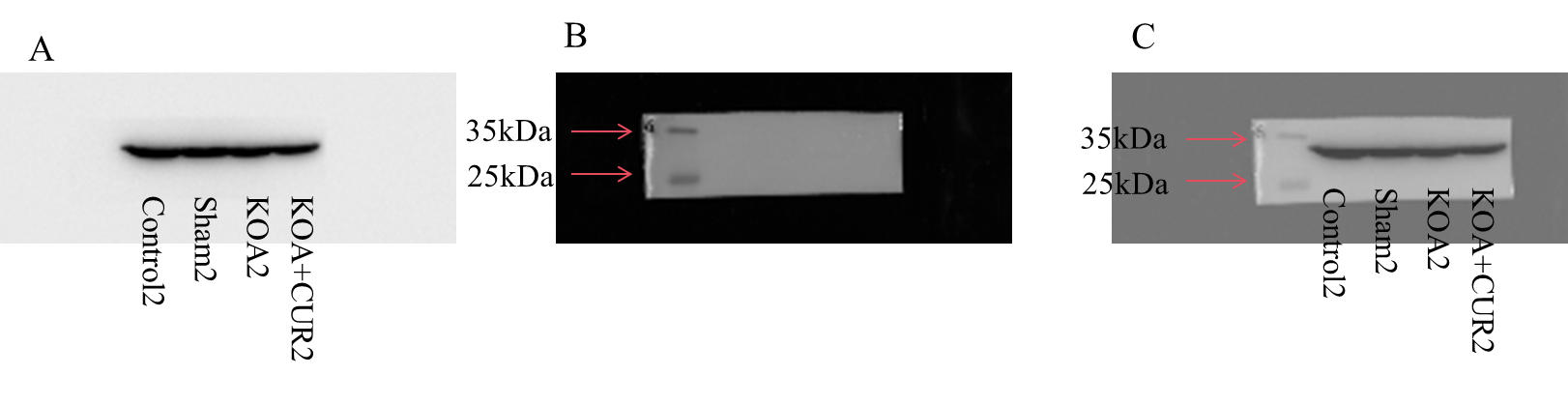


Original blot images of GAPDH. The blot (A) we used in Figure 5.

(A) Blot image of GAPDH.

(B) Protein marker image of GAPDH.

(C) Blot image of GAPDH (A) merged with protein marker image (B).

Figure S7


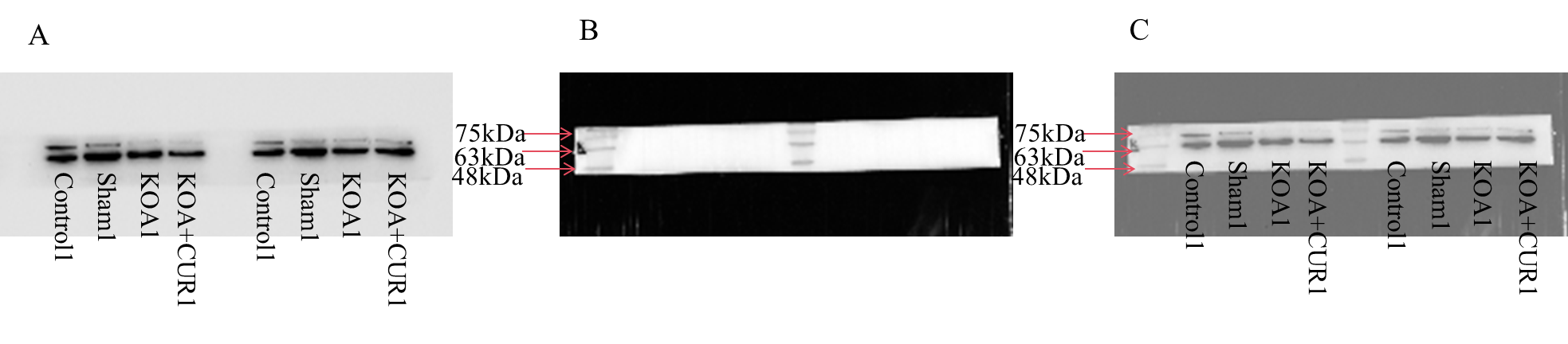


Original blot images of P62. The right side of the blot (A) we used in Figure 6.

(A) Blot image of P62.

(B) Protein marker image of P62.

(C) Blot image of P62 (A) merged with protein marker image (B).

Figure S8


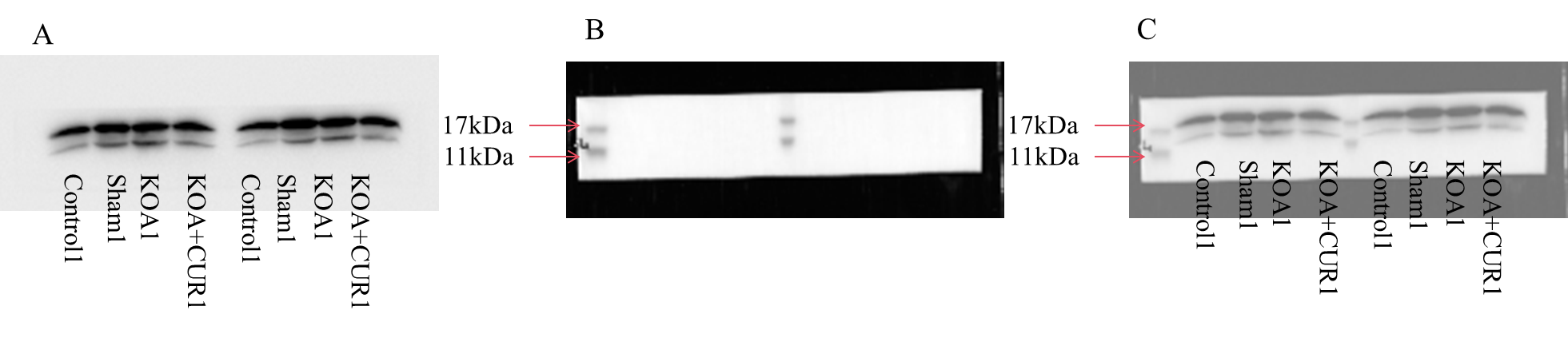


Original blot images of LC3. The left side of the blot (A) we used in Figure 6.

(A) Blot image of LC3.

(B) Protein marker image of LC3.

(C) Blot image of LC3 (A) merged with protein marker image (B).

Figure S9


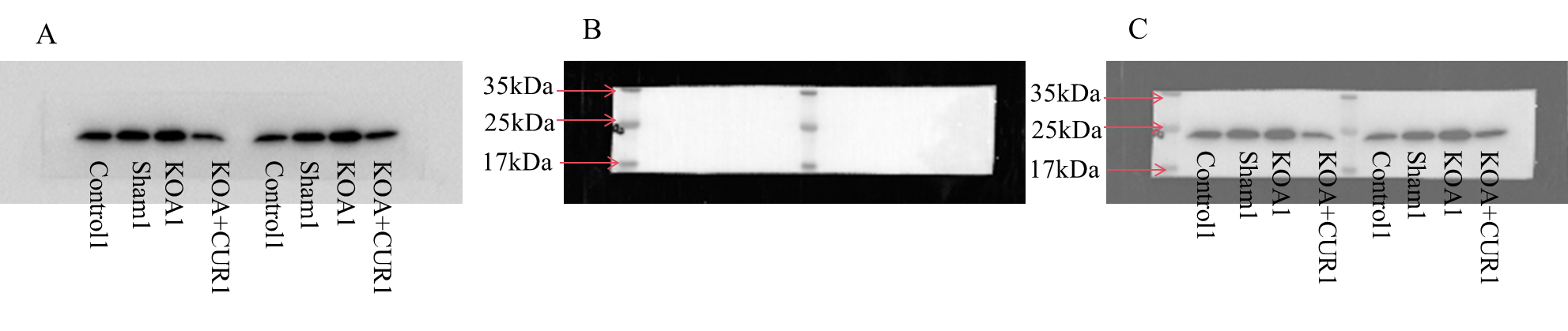


Original blot images of Ac-SOD2. The right side of the blot (A) we used in Figure 6.

(A) Blot image of Ac-SOD2.

(B) Protein marker image of Ac-SOD2.

(C) Blot image of Ac-SOD2 (A) merged with protein marker image (B).

Figure S10


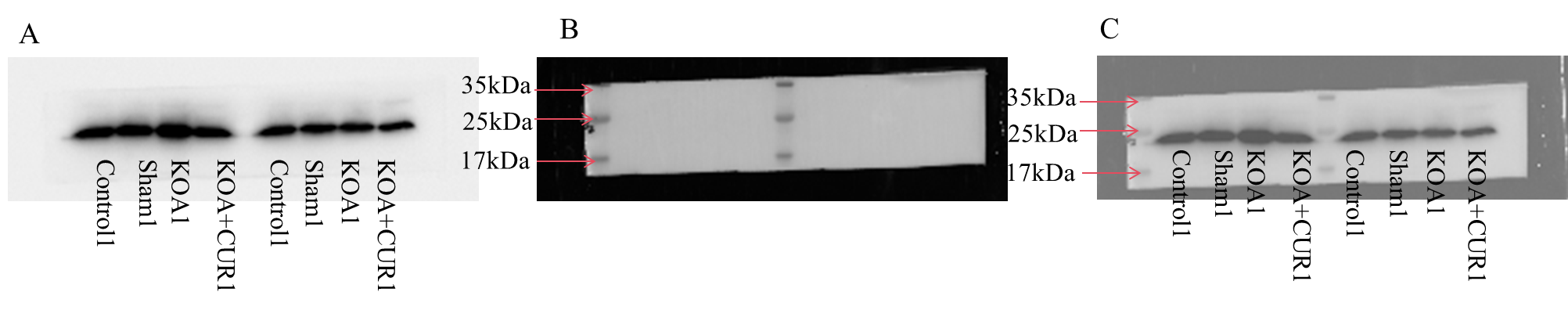


Original blot images of SOD2. The right side of the blot (A) we used in Figure 6.

(A) Blot image of SOD2.

(B) Protein marker image of SOD2.

(C) Blot image of SOD2 (A) merged with protein marker image (B).

Figure S11


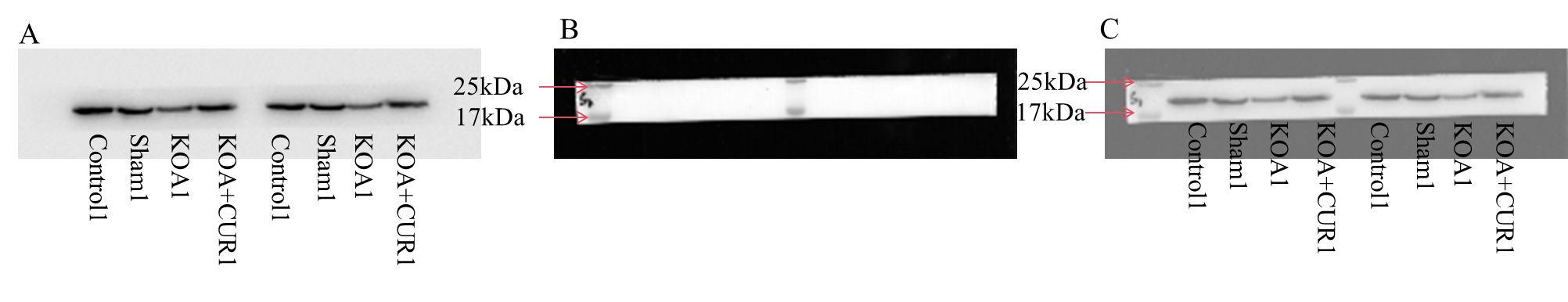


Original blot images of SIRT3. The left side of the blot (A) we used in Figure 6.

(A) Blot image of SIRT3.

(B) Protein marker image of SIRT3.

(C) Blot image of SIRT3 (A) merged with protein marker image (B).

Figure S12


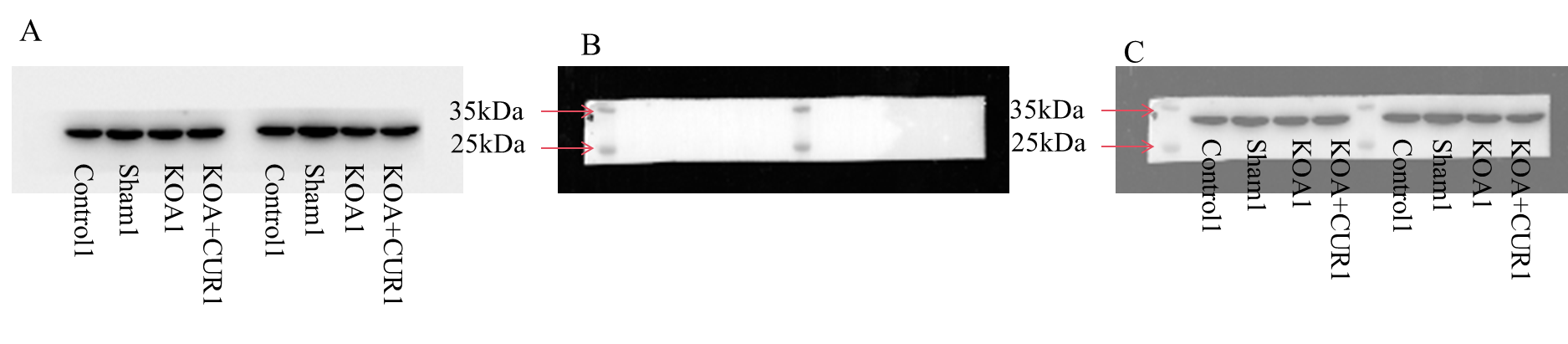


Original blot images of GAPDH. The right side of the blot (A) we used in Figure 6.

(A) Blot image of GAPDH.

(B) Protein marker image of GAPDH.

(C) Blot image of GAPDH (A) merged with protein marker image (B).

**The following images of the original blots were from replicate western blotting experiments using different rats. Not presented in the manuscript.**

Figure S13


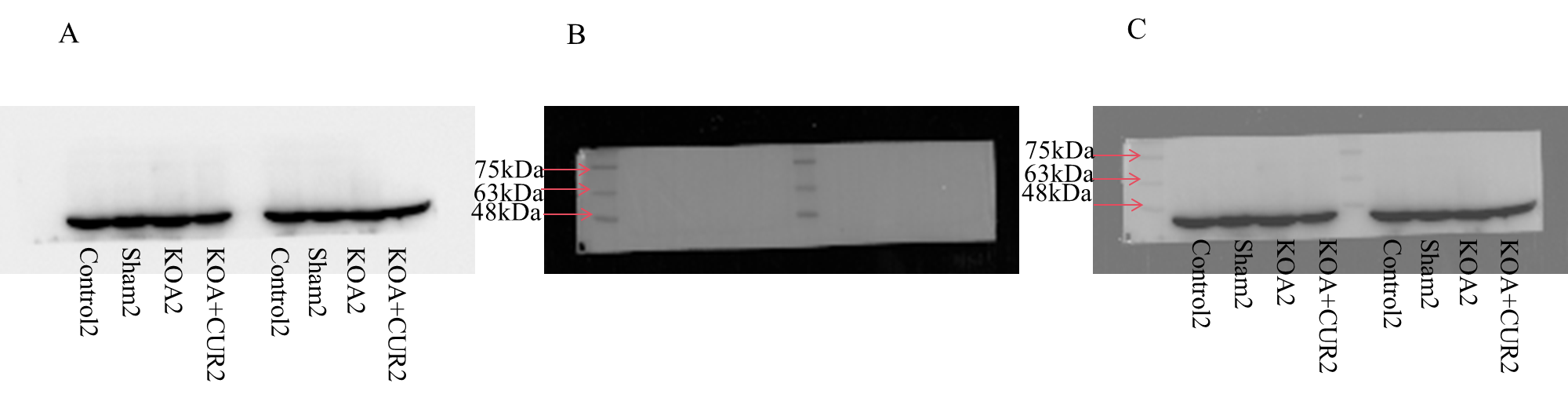


Original blot images of Atrogin-1.

(A) Blot image of Atrogin-1.

(B) Protein marker image of Atrogin-1.

(C) Blot image of Atrogin-1 (A) merged with protein marker image (B).

Figure S14


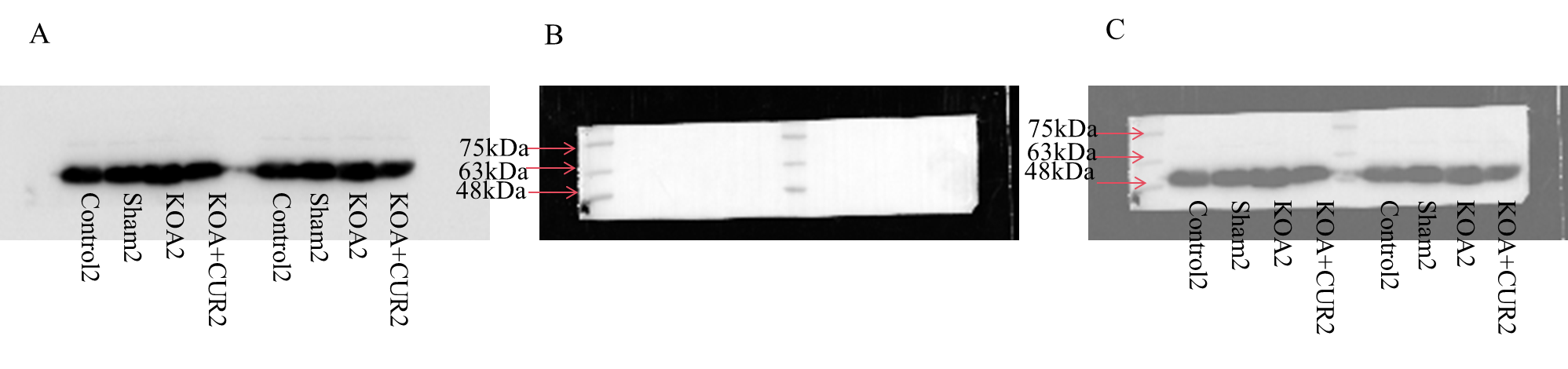


Original blot images of MuRF-1.

(A) Blot image of MuRF-1.

(B) Protein marker image of MuRF-1.

(C) Blot image of MuRF-1 (A) merged with protein marker image (B).

Figure S15


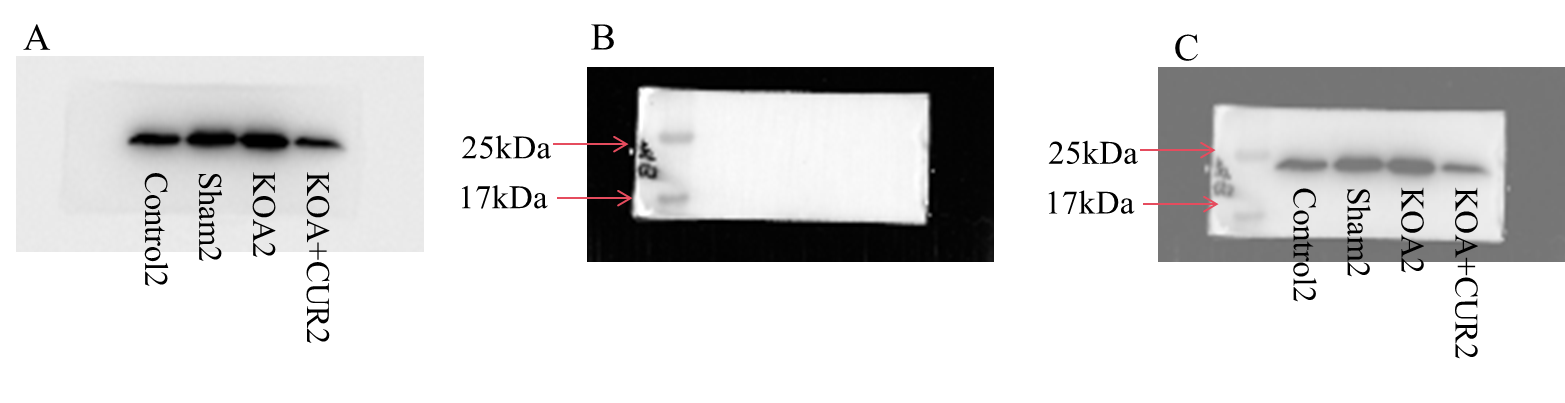


Original blot images of Ac-SOD2.

(A) Blot image of Ac-SOD2.

(B) Protein marker image of Ac-SOD2.

(C) Blot image of Ac-SOD2 (A) merged with protein marker image (B).

Figure S16


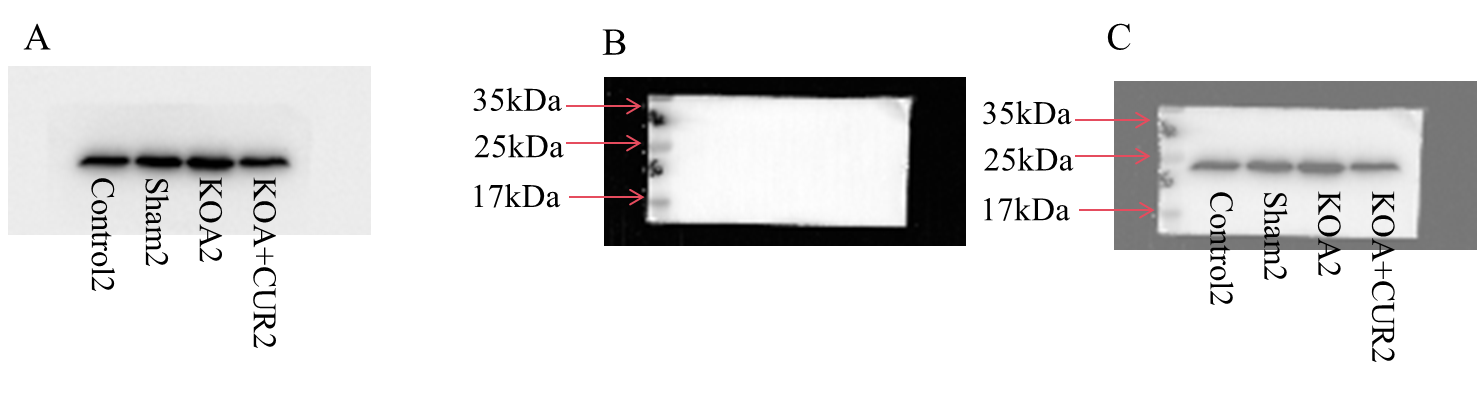


Original blot images of SOD2.

(A) Blot image of SOD2.

(B) Protein marker image of SOD2.

(C) Blot image of SOD2 (A) merged with protein marker image (B).

Figure S17


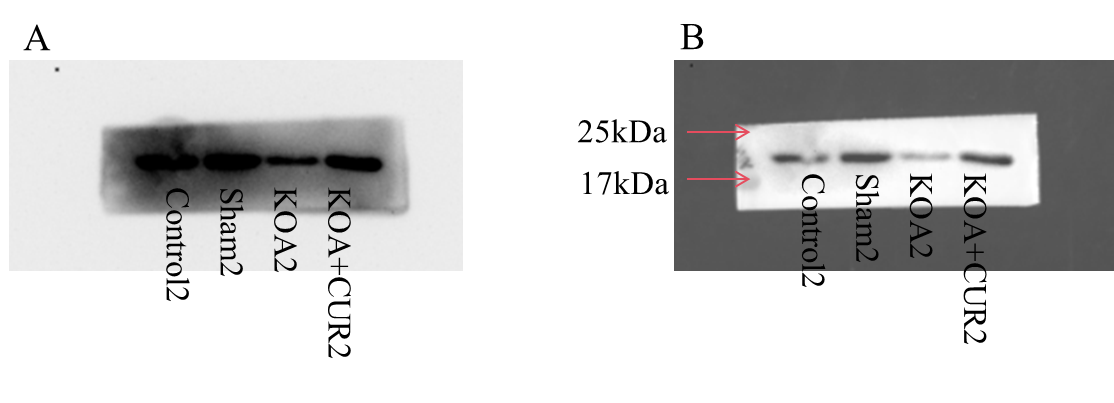


Original blot images of SIRT3.

(A) Blot image of SIRT3.

(B) Protein marker image of SIRT3.

(C) Blot image of SIRT3 (A) merged with protein marker image (B).

Figure S18


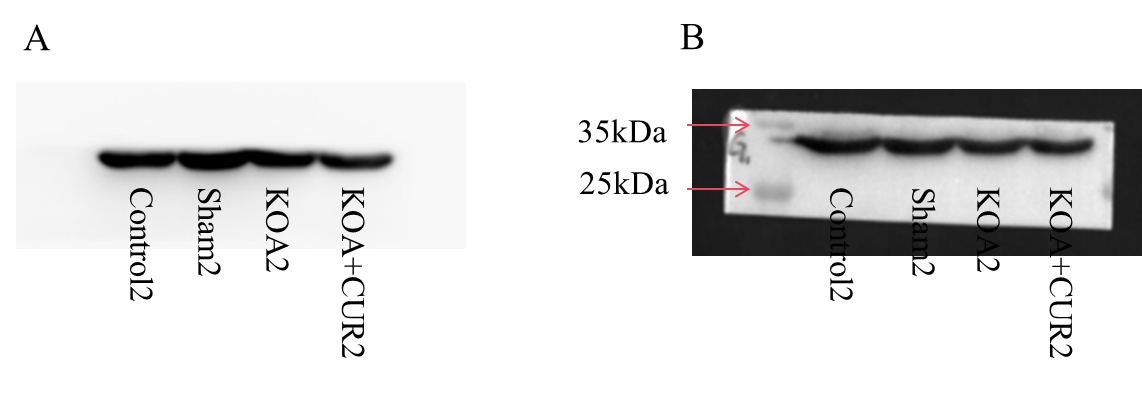


Original blot images of GAPDH.

(A) Blot image of GAPDH.

(B) Protein marker image of GAPDH.

(C) Blot image of GAPDH (A) merged with protein marker image (B).

**The following images of the original blots were from replicate western blotting experiments using different rats. Not presented in the manuscript.**

Figure S19


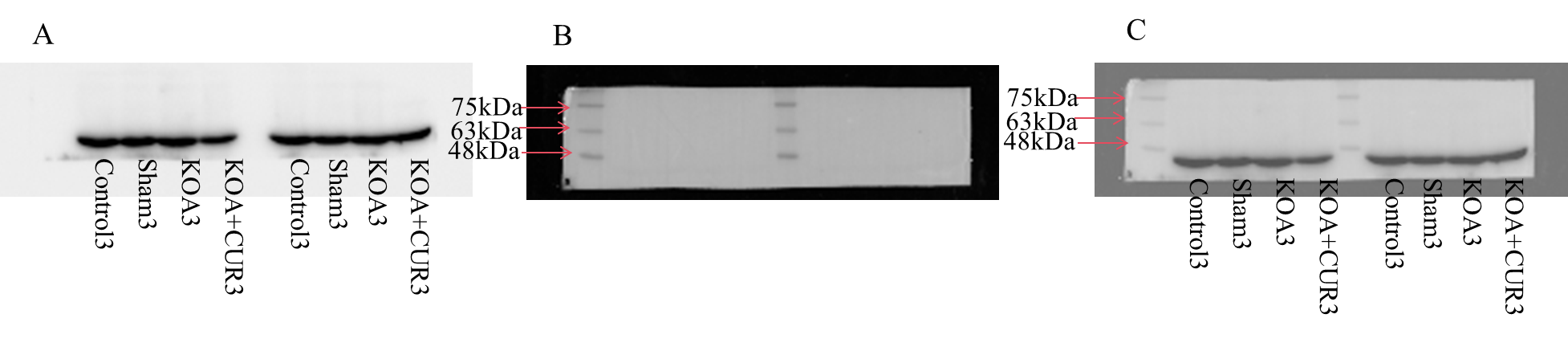


Original blot images of Atrogin-1.

(A) Blot image of Atrogin-1.

(B) Protein marker image of Atrogin-1.

(C) Blot image of Atrogin-1 (A) merged with protein marker image (B).

Figure S20


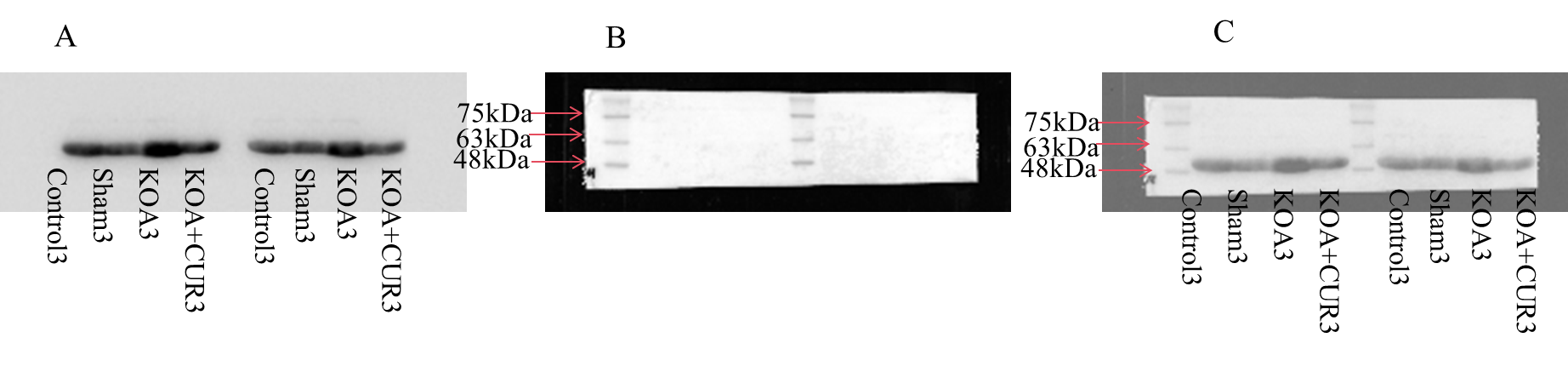


Original blot images of MuRF-1.

(A) Blot image of MuRF-1.

(B) Protein marker image of MuRF-1.

(C) Blot image of MuRF-1 (A) merged with protein marker image (B).

Figure S21


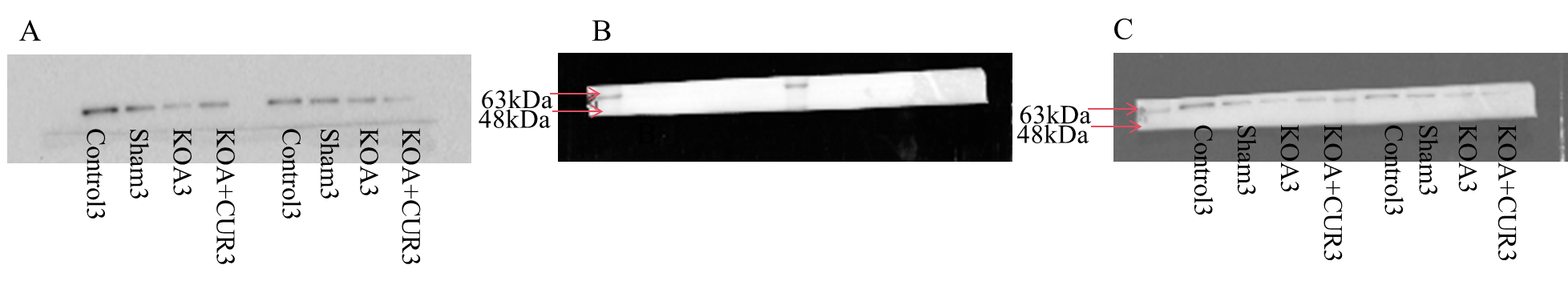


Original blot images of P62.

(A) Blot image of P62.

(B) Protein marker image of P62.

(C) Blot image of P62 (A) merged with protein marker image (B).

Figure S22


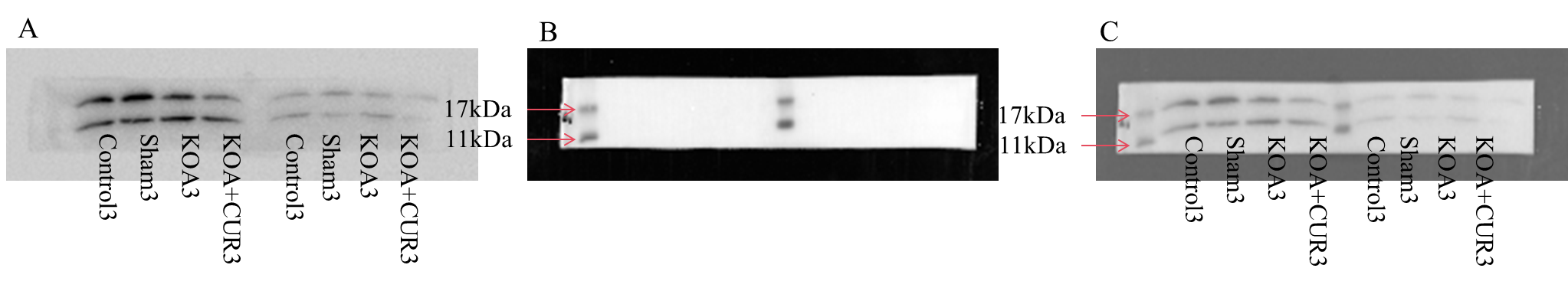


Original blot images of LC3.

(A) Blot image of LC3.

(B) Protein marker image of LC3.

(C) Blot image of LC3 (A) merged with protein marker image (B).

Figure S23


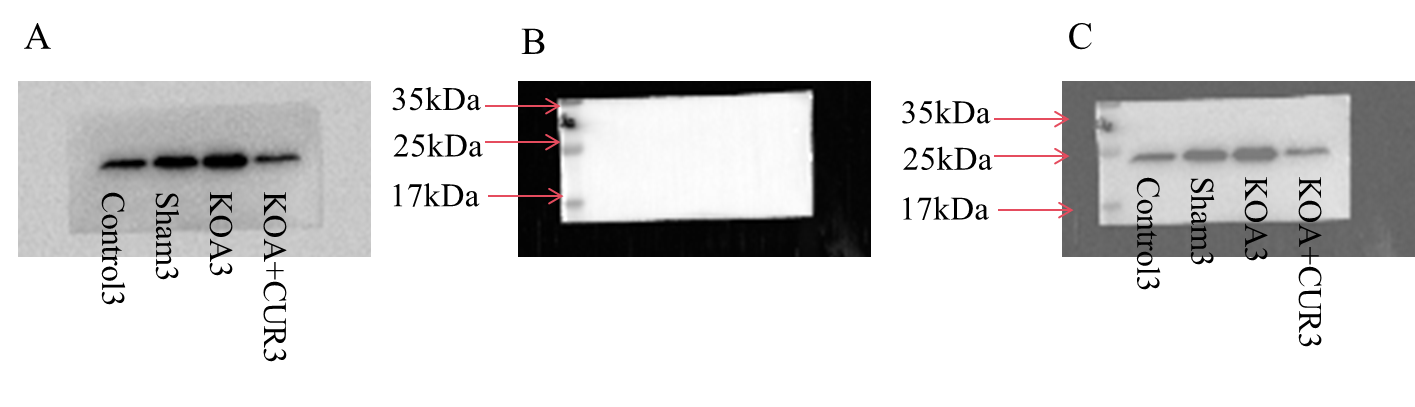


Original blot images of Ac-SOD2.

(A) Blot image of Ac-SOD2.

(B) Protein marker image of Ac-SOD2.

(C) Blot image of Ac-SOD2 (A) merged with protein marker image (B).

Figure S24


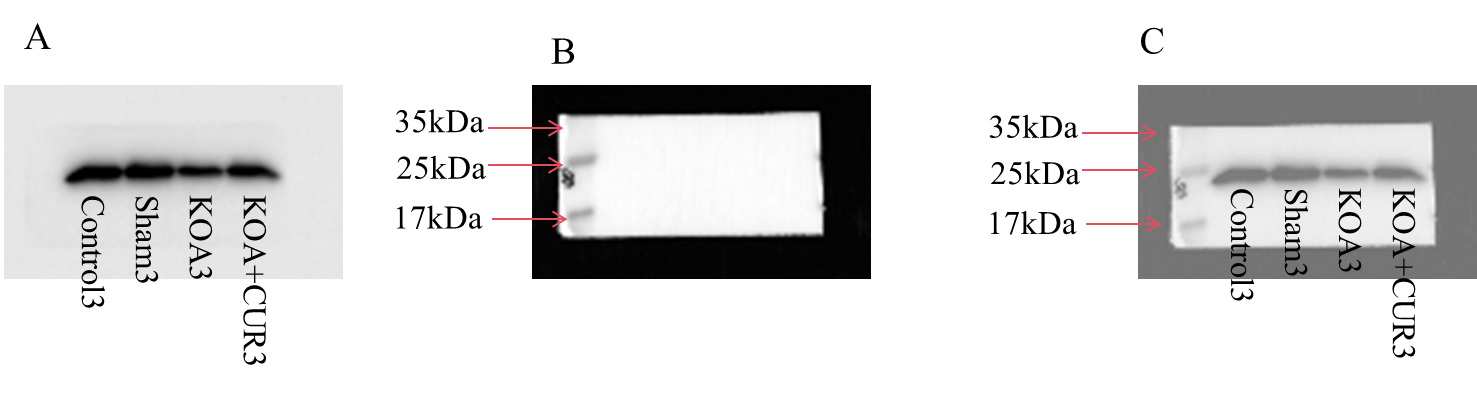


Original blot images of SOD2.

(A) Blot image of SOD2.

(B) Protein marker image of SOD2.

(C) Blot image of SOD2 (A) merged with protein marker image (B).

Figure S25


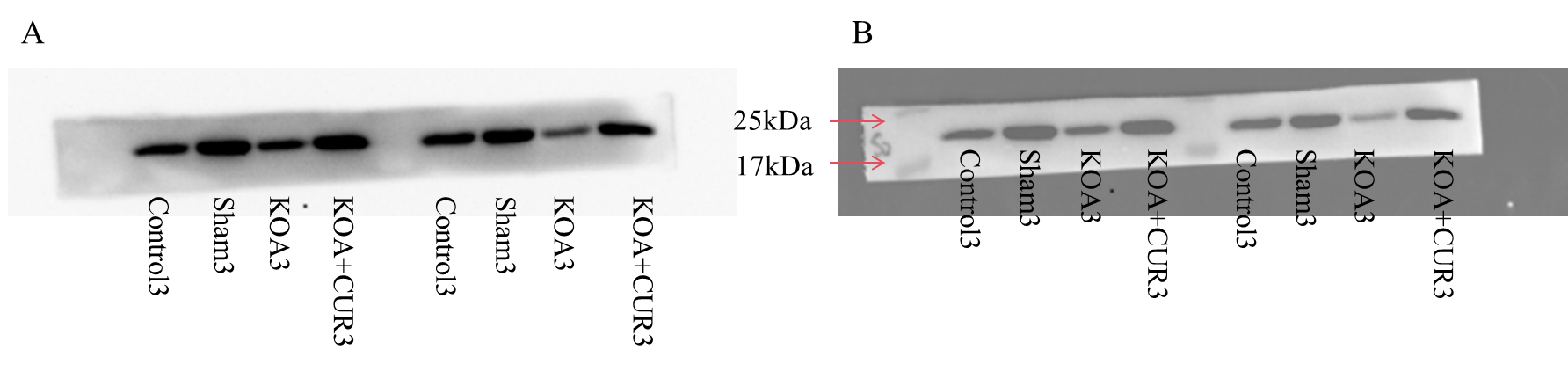


Original blot images of SIRT3.

(A) Blot image of SIRT3.

(B) Protein marker image of SIRT3.

(C) Blot image of SIRT3 (A) merged with protein marker image (B).

Figure S26


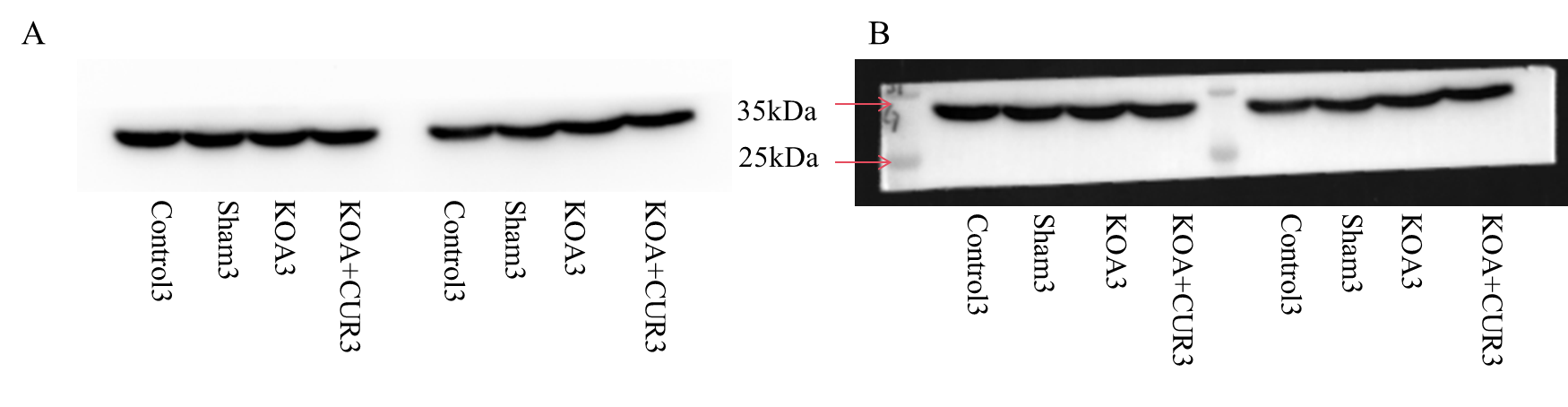


Original blot images of GAPDH.

(A) Blot image of GAPDH.

(B) Protein marker image of GAPDH.

(C) Blot image of GAPDH (A) merged with protein marker image (B).

The brand and item number of the ColorMixed Protein Marker we used is PR1910, Solarbio, China.


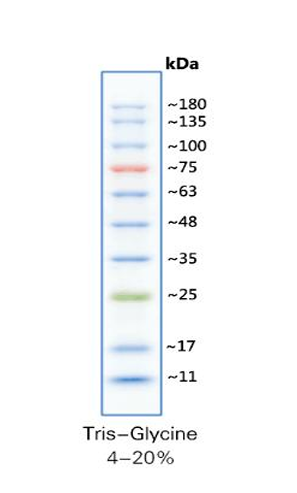

Supplement: Supplementary file 1 — Supplementary Figures. [file 41598_2024_58375_MOESM1_ESM.docx]
